# Supplementary figures and images for: The OASIS walking study—Older adults with cognitive impairment performing sit to stands and walking in transitional care programs: Protocol for a feasibility study
Source: PLoS One. 2024 Sep 16;19(9):e0308268. doi: 10.1371/journal.pone.0308268 (PMC11404812; doi:10.1371/journal.pone.0308268)

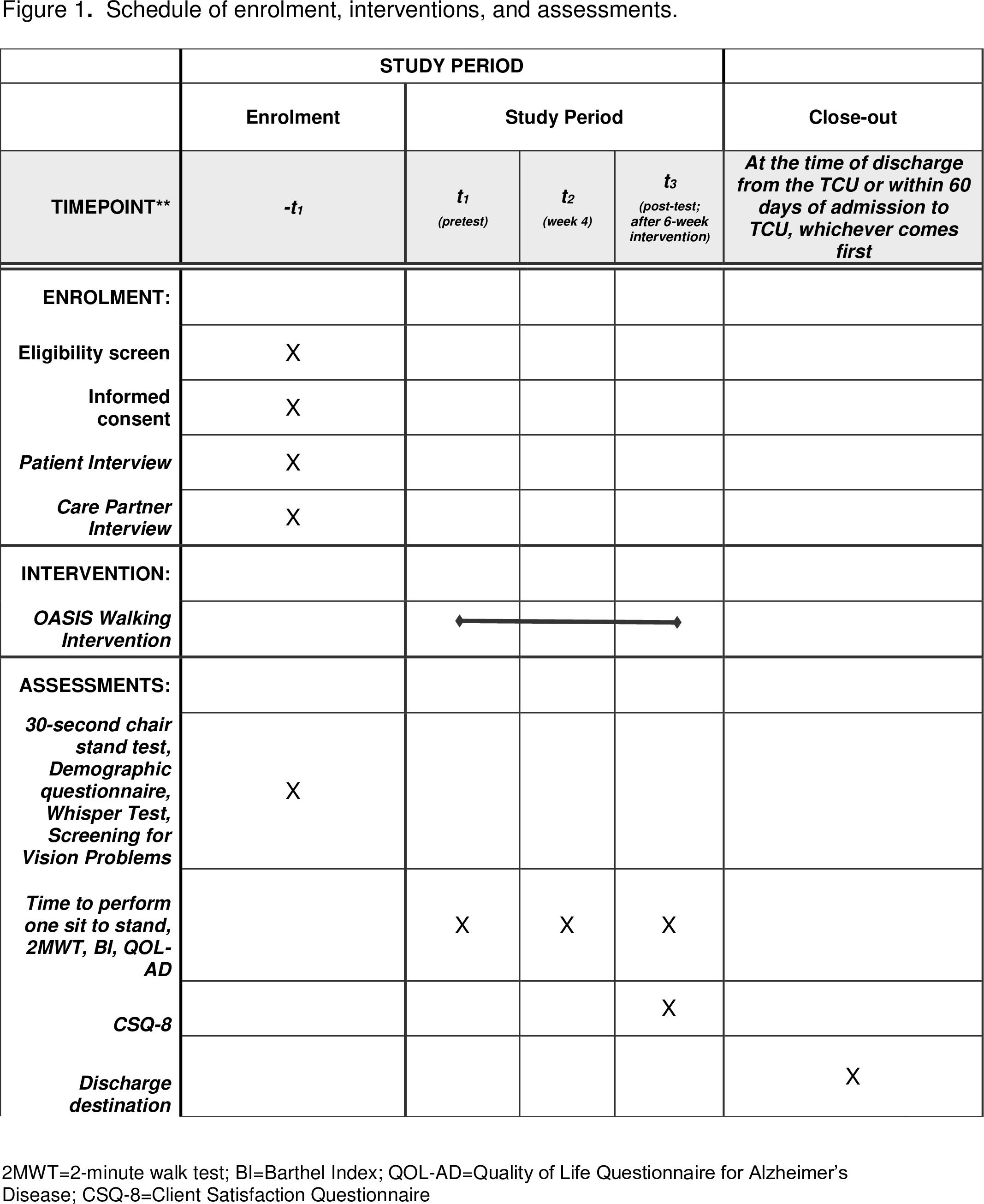

Supplement: S1 Fig — (TIF) [file pone.0308268.s002.tif]

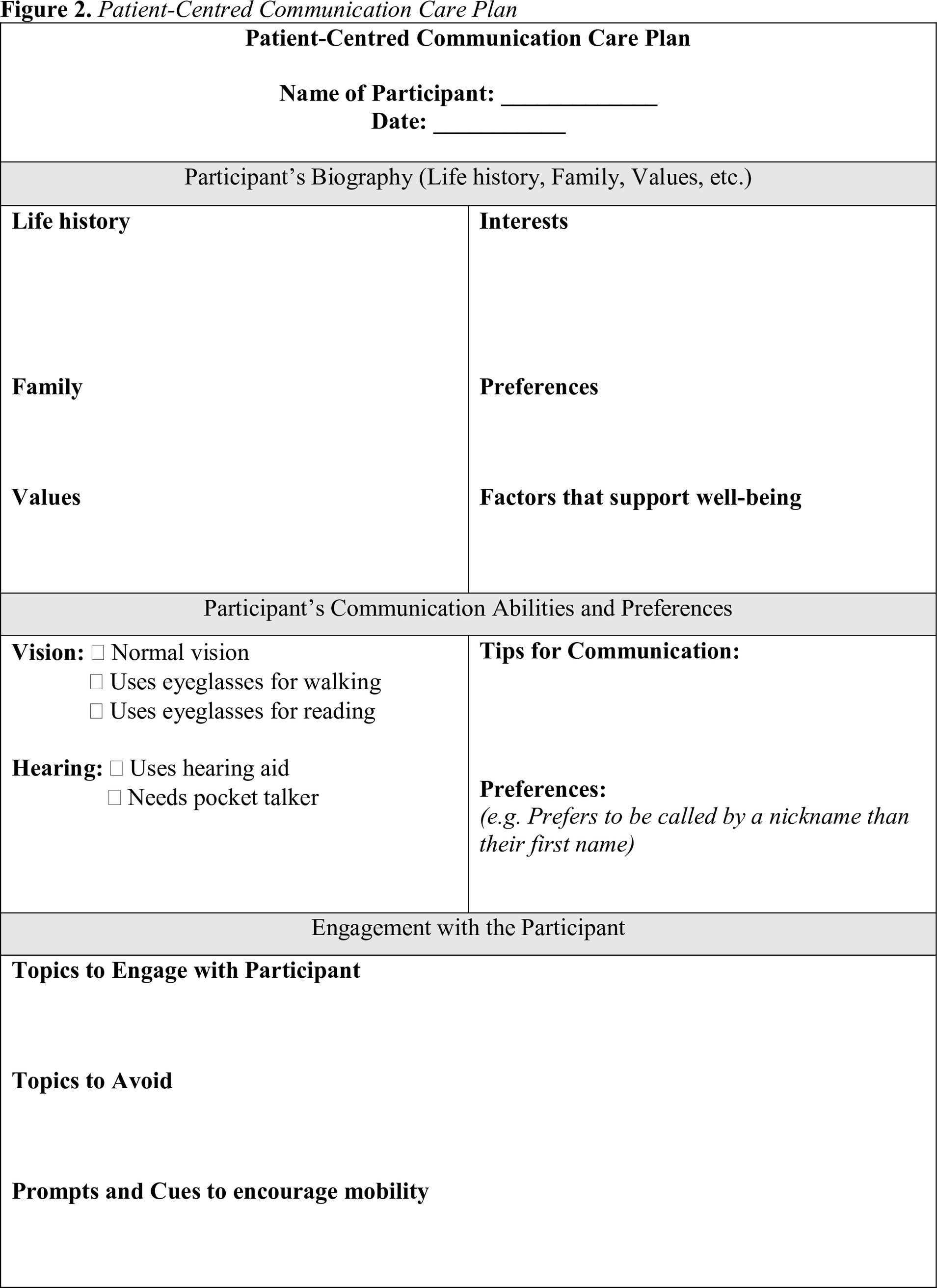

Supplement: S2 Fig — (TIF) [file pone.0308268.s003.tif]
