# Supplementary material for: The OASIS walking study—Older adults with cognitive impairment performing sit to stands and walking in transitional care programs: Protocol for a feasibility study
Source: PLoS One. 2024 Sep 16;19(9):e0308268. doi: 10.1371/journal.pone.0308268 (PMC11404812; doi:10.1371/journal.pone.0308268)
Supplement: S2 Appendix — (DOCX) [file pone.0308268.s005.docx]

**S2 Appendix - Consent Form to Participate in a Research Study**-Participant/SDM

**Study title:**  The OASIS Walking Study – **O**lder **A**dults with cognitive impairment performing **Si**t to **S**tands and **Walking** in transitional care programs

**Investigators:** Dr. Katherine McGilton, RN, PhD

**Contact information:** email: [kathy.mcgilton@uhn.ca](mailto:kathy.mcgilton@uhn.ca); phone #: 416-597-3422 ext 2500

Please note that communication via e-mail is not absolutely secure. Thus, please do not communicate personal sensitive information via e-mail.

**Funding:** This study is funded by a grant from the Canadian Institute of Health Research (CIHR)

Disclosure: This study is a graduate thesis project of Alexia Cumal, RN, PhD Candidate.

**Introduction:**

This Consent Form is addressed to the patient. However, in the case that the patient does not have capacity to provide informed consent for themselves the form is given to you as their substitute decision maker for whom informed consent will be obtained for participating in the study.

You are being asked to take part in a research study. Please read the information about the study presented in this form. The form includes details on the study’s risks and benefits that you should know before you decide if you would like to take part. You should take as much time as you need to make your decision. You should ask the study investigator or study staff to explain anything that you do not understand and make sure that all of your questions have been answered before signing this consent form. Before you make your decision, feel free to talk about this study with anyone you wish including your friends and family. Participation in this study is voluntary.

**Background and Purpose:**

Many older adults with cognitive impairment who are ready to leave the hospital cannot do so because there are no homecare services or residential/long-term care placements available to them. Transitional care programs (TCPs) are a recently introduced innovation to provide services that these individuals need to recover in their home, residential or long-term care homes. We conducted a review study which found that older adults with cognitive impairment do not fare as well as those without cognitive impairment in TCPs. However, in the nursing home setting, walking programs have resulted in significant improvements in outcomes for older adults with cognitive impairment. Sit to stand activity has also resulted in improved outcomes for older adults with cognitive impairment in nursing homes. And so, we developed a mobility intervention (walking program with sit to stand activity) that aims to improve their outcomes.

The purpose of this study is to determine if it is feasible to carry out a mobility intervention – called the **OASIS Walking Intervention** (**O**lder **A**dults performing **Si**t to **S**tands and **Walking**) in TCPs and to see if they are able to improve patient outcomes.

This intervention is not the standard of care provided on the unit.

Standard of care on the unit includes: For patients in the long-term care stream: one-to-one sessions 2-3 times per week of strengthening and balance exercises. For patients in the Rehab stream, one-to-one sessions 5 times per week of strengthening, balance, and may include some walking and group therapy 5 times per week.

This intervention will not affect the standard of care, and so patients who participate in this study will also continue to receive the standard of care.

You are being asked to participate in the study because you are a patient in a Transitional Care Program (TCP) with cognitive impairment.

Up to 26 patients (from two transitional care units in Ontario) and their care partners will participate in this study and it will take about 8 months to complete.

**Study visits and procedures:**

If you agree to participate, you will be asked over the course of the study to participate in several activities. The timing and description of the study commitments are below:

- **Admission Interview and Assessment:** You will be asked to meet the principal investigator (PI) of the study, who will ask you some questions about your interests, life history, habits, and preferences. The interview will take about **30 minutes** and will take place in person. The interview will be audio recorded. The recording and the transcript will be saved to the University Health Network’s server. After the interview, the transcript will be reviewed for accuracy and any personal information will be removed. The recording will then be deleted. You will be asked if you have any vision problems and if you need to use eye glasses. There will be a quick test on hearing, where a research member will ask you to repeat a set of 3 numbers with a normal voice and with a whispered voice. Your ability to walk and your ability to stand from sitting will be assessed. This should take about **15 minutes**.
- **Interview with your family member (substitute decision maker)**: The interventionist I will call your family member to ask them some questions about you, including how well you were able to walk and do things at home before you came to the hospital. The interview will take about **45 minutes**.
- **Care plan:** Based on your admission interview and the interview with your family member, the interventionist will create a care plan, which will be referred to during the study, and which will be shared with your care team at the TCU after the study.
- **Outcome Measurement:** A member of the research team will assess your walking and ability to stand from sitting two more times during the study (once in the middle of the study (after 3 weeks) and once at the end of the study (after 6 weeks)) and ask you about your quality of life. The first time, you will be asked demographic questions. The third time you will be asked about your satisfaction with the walking program. The first time should take about **37 minutes**, the second time should take about **29 minutes** and the third time should take about 45 minutes. The interventionist will assess your ability to perform activities of daily living and will ask a nurse/care staff for more details if required three times during the study.

**Activity Sessions:** The interventionist will meet with you for about **45 minutes**, **5 days a week for 6 weeks.** During each session, the interventionist will walk with you down the hallways of the unit for as long as you are able to tolerate, for up to 30 minutes each session. You can take rest breaks as needed. The interventionist will chat with you during the session, either as you are walking and/or during rest breaks, based on what you prefer. As time goes on, the interventionist may ask you to walk longer distances and/or a little bit faster, based on how well you are walking. The interventionist will also ask you to stand from the seated position a number of times, based on the target number of sit to stands that is determined at the beginning of the study, for up to 15 minutes. If needed, the interventionist will ask you to watch them do the activity and ask you to do the activity.

- **Chart review: At the beginning of the study,** the interventionist will review your medical records for information on your past medical history. At the end of the study, the interventionist will review your records to see where you are discharged to after the TCP.
- If at any point during the study, you don’t want to continue, ask to stop. If you experience discomfort, say something and/or ask to stop.

| **Time** | **Name of Test/Procedure** | **Time to Conduct Test/Procedure** |
| --- | --- | --- |
| Before intervention sessions | - Interview with the interventionist and the Participant about your interests, life history, habits, and preferences | 30 minutes |
|  | - Vision Screening - Whispered Voice Test (Hearing test) | 15 minutes |
| Time 1 (Before intervention sessions) | - Demographic Questionnaire (age, sex, gender), - Chair stand tests (Time to perform one sit to stand, 30 second chair stand) - Two-minute walk test - Quality of Life Questionnaire | 37 minutes |
| Time 2 (After 3 weeks) | - Chair stand test (Time to perform one sit to stand) - Two-minute walk test - Quality of Life Questionnaire | 29 minutes |
| Time 3 (After 6 weeks) | - Chair stand test (Time to perform one sit to stand) - Two-minute walk test - Quality of Life Questionnaire - Satisfaction  Questionnaire | 44 minutes |

**Study Assessments Involving Tests or Interviews with the Participant**

**Potential Risks*.***

Taking part in this study has risks. Some of these risks we know about. There is also a possibility of risks that we do not know about and have not been seen in humans to date. Please call the study doctor if you have any side effects even if you do not think it has anything to do with this study.

The risks we know of are:

- Any study involving walking and activity comes with a risk for falls. For this study, we are taking precautions to reduce the risk of falls by having two people with you at each activity session for the first week and in following weeks if the physiotherapist recommends it– the interventionist will walk beside you and the research assistant will follow behind you with a wheelchair in case you feel tired.
- Soreness of leg muscles or feeling tired. If you feel any of these symptoms, let the interventionist know, and you can take rest breaks as often as you need.

**Potential Benefits:**

You may not receive direct benefit from this study. Information learned from this study may also help to refine the activities to be done with future older adult patients with cognitive impairment in TCPs.

**Alternatives to Being in the Study:**

The alternative to participation in this study is to receive the usual care that is provided on the unit.

**Confidentiality:**

Your data will be shared as described in this consent form or as required by law. All personal information such as your name will be removed from the data and will be replaced with a number. A list linking the number with your name will be kept by the study investigator in a secure place, separate from your file.

**Personal Health Information**

If you agree to participate in this study, the research team will look at your personal health information and collect only the information they need for the study. Personal health information is any information that could identify you and includes your:

- Name
- Age
- Past medical history

The following people may come to the hospital or be given remote access to an electronic portal (via the internet) to look at the study records and at your personal health information to check that the information collected for the study is correct and to make sure the study is following proper laws and guidelines. When using the electronic portal, we will share your file identified by a numeric code using a secure method, so that your records are included as part of their review.

- Representatives of the University Health Network (UHN) including the UHN Research Ethics Board

These individuals have completed privacy training and signed confidentiality agreements and/or are required by law to keep your information confidential.

Whether on-site or remotely, UHN makes all efforts to ensure that your information is shared in a way that is secure and private (encrypted). However, any electronic communication carries some risk of third parties gaining unauthorized access to information.

The research team will keep any personal health information about you in a secure and confidential location for 10 years.

Your participation in this study will also be recorded in your medical record at this hospital. This is for clinical safety purposes.

**Research Information in Shared Clinical Records**

If you participate in this study, information about you from this research project may be stored in your hospital file and in the UHN computer system. The UHN shares the patient information stored on its computers with other hospitals and health care providers in Ontario so they can access the information if it is needed for your clinical care. The study team can tell you what information about you will be stored electronically and may be shared outside of the UHN. If you have any concerns about this, or have any questions, please contact the UHN Privacy Office at 416-340-4800, x6937 (or by email at privacy@uhn.ca).

**Study Information that Does Not Identify You**

You will not be named in any reports, publications, or presentations that may come from this study. The interview with participants and care partners at the beginning of the study will be audio recorded and then transcribed. All transcripts will be de-identified (names will be removed), and audio recordings will be destroyed upon transcription.

Unidentified quotes obtained during the interview may be used in the sharing of this research.

**Voluntary participation:**

Your participation in this study is voluntary. You may decide not to be in this study, or to be in the study now, and then change your mind later. You may leave the study at any time, your decision will not affect your care. We will give you new information that is learned during the study that might affect your decision to stay in the study.

You may refuse to answer any question you do not want to answer, or not answer an interview question by saying “pass”.

**Withdrawal from the study:**

The Researchers can take you off the study if you are not able to follow study procedure.

You may withdraw from the study at any time. If you decide to leave the study, you have the right to request withdrawal of information collected about you, including the interview information. Information can be withdrawn at anytime, even once analysis has started. You may also withdraw but allow the study team to continue use of the data that has already been collected and not to collect new information. Let the research team know.

**Cost and reimbursement:**

You will not have to pay for any procedures involved in this study. As a token of appreciation and in recognition of your time and effort, a $5 gift card to a coffee shop will be given to you at each of the three outcome measurement stages of the research. By the end of the study, you will be given a total of $15 in gift cards.

**Rights as a participant:**

By signing this form, you do not give up any of your legal rights against the investigators, sponsor or involved institutions for compensation, nor does this form relieve the investigators, sponsor or involved institutions of their legal and professional responsibilities.

If you are harmed as a direct result of taking part in this study, all necessary medical treatment will be made available to you at no cost.

**Conflict of interest:**

Researchers have an interest in completing this study. Their interests should not influence your decision to participate in this study.

**Questions about the study:**

If you have any questions, concerns or would like to speak to the study team for any reason, please call: Dr. Katherine McGilton at 416-597-3422 ext 2500.

If you have any questions about your rights as a research participant or have concerns about this study, call the Chair of the University Health Network Research Ethics Board (UHN REB) or the Research Ethics office number at 416-581-7849. The REB is a group of people who oversee the ethical conduct of research studies. The UHN REB is not part of the study team. Everything that you discuss will be kept confidential.

You will be given a signed copy of this consent form.

**Consent:**

This study has been explained to me and any questions I had have been answered.

I know that I may leave the study at any time. I agree to the use of my information as described in this form. I agree to take part in this study.

Print Study Participant’s Name Signature Date

**Substitute Decision Maker:**

- After considering the wishes, values, and goals of the patient they would permit the study team to perform study procedures and data collection. I can reverse this decision at any time. The study team will review this consent with the patient when their capacity for consent is regained.

Name of Substitute Decision Maker Signature Date

Relationship to Participant

My signature means that I have explained the study to the participant named above. I have answered all questions.

Print Name of Person Signature Date Obtaining Consent

The consent form was read to the participant. The person signing below attests that the study as set out in this form was accurately explained to the participant and has had any questions answered.

_____________________

Print Name of Witness Signature Date

Relationship to Participant
