# Supplementary material for: The OASIS walking study—Older adults with cognitive impairment performing sit to stands and walking in transitional care programs: Protocol for a feasibility study
Source: PLoS One. 2024 Sep 16;19(9):e0308268. doi: 10.1371/journal.pone.0308268 (PMC11404812; doi:10.1371/journal.pone.0308268)
Supplement: S3 Appendix — (DOCX) [file pone.0308268.s006.docx]

**S3 Appendix**

**Consent Form to Participate in a Research Study**

**Study title:**  The OASIS Walking Study – **O**lder **A**dults with cognitive impairment performing **Si**t to **S**tands and **Walking** in transitional care programs-Care Partner

**Investigators:** Dr. Katherine McGilton, RN, PhD

**Contact information:** email: [kathy.mcgilton@uhn.ca](mailto:kathy.mcgilton@uhn.ca); phone #: 416-597-3422 ext 2500

Please note that communication via e-mail is not absolutely secure. Thus, please do not communicate personal sensitive information via e-mail.

**Funding:** This study is funded by a grant from the Canadian Institute of Health Research (CIHR)

Disclosure: This study is a graduate thesis project of Alexia Cumal, RN, PhD Candidate.

**Introduction:**

You are being asked to take part in a research study. Please read the information about the study presented in this form. The form includes details on study’s risks and benefits that you should know before you decide if you would like to take part. You should take as much time as you need to make your decision. You should ask the study investigator or study staff to explain anything that you do not understand and make sure that all of your questions have been answered before signing this consent form.  Before you make your decision, feel free to talk about this study with anyone you wish including your friends and family. Participation in this study is voluntary.

**Background and Purpose:**

Many older adults with cognitive impairment who are ready to leave the hospital cannot do so because there are no homecare services or residential/long-term care placements available to them. Transitional Care Programs (TCPs) are a recently introduced innovation to provide services that these individuals need to recover in their home, residential or long-term care homes. We conducted a review study which found that older adults with cognitive impairment do not fare as well as those without cognitive impairment in TCPs, and so we developed a mobility intervention that aims to improve their outcomes.

The purpose of this study is to determine if it is feasible to carry out a mobility intervention – called the **OASIS Walking Intervention** (**O**lder **A**dults performing **Si**t to **S**tands and **Walking**) in TCPs and to see if they are able to improve patient outcomes.

You are being asked to participate in the study as a care partner of a patient with cognitive impairment in a Transitional Care Program (TCP).

Up to 26 patients from one transitional care unit in Ontario and their care partners will participate in this study and it will take about 8 months to complete.

**Study procedures:**

If you agree to participate, you will be asked to participate in one interview. The purpose of the interview is to learn about the participant, their interests, history, your experiences and the experiences of someone you know staying on the TCP unit. The interview will last approximately 45 minutes and will be conducted by a member of the research team in person or over the phone at a time convenient for you. In addition, prior to the interview you will be asked to complete a demographic questionnaire (approximately 10 minutes to complete) sharing information such as your age, sex, gender, ethnicity, relationship to the person staying on the TCP unit, and how often you see the patient on the TCP unit. The interview will be audio recorded and transcribed verbatim. Audio recordings are necessary for this study, as they are required to ensure reliable analysis.

**Risks:**

Taking part in this study has risks. Some of these risks we know about. There is also a possibility of risks that we do not know about and have not been seen in humans to date. Please call the study doctor if you have any side effects even if you do not think it has anything to do with this study.

The risks we know of are:

- Answering some interview question may make you feel uncomfortable. You may refuse to answer any question you do not want to answer

**Benefits:**

You may not receive direct benefit from being in this study. Information learned will help to inform the patient-centered communication care plan for your loved one, which will be used during the mobility intervention.

**Confidentiality:**

Your data will be shared as described in this consent form or as required by law. All personal information such as your name will be replaced with a number. A list linking the number with your name will be kept by the study doctor in a secure place, separate from your file.

**Personal Health Information**

If you agree to participate in this study, the research team will look at your personal health information and collect only the information they need for the study. Personal health information is any information that could identify you and includes your:

- Name
- Phone Number

The following people may come to the hospital or be given remote access to an electronic portal (via the internet) to look at the study records and at your personal health information to check that the information collected for the study is correct and to make sure the study is following proper laws and guidelines. When using the electronic portal, we will share your file identified by a numeric code using a secure method, so that your records are included as part of their review.

- Representatives of the University Health Network (UHN) including the UHN Research Ethics Board

These individuals have completed privacy training and signed confidentiality agreements and/or are required by law to keep your information confidential.

Whether on-site or remotely, UHN makes all efforts to ensure that your information is shared in a way that is secure and private (encrypted). However, any electronic communication carries some risk of third parties gaining unauthorized access to information.

The research team will keep any personal health information about you in a secure and confidential location for 10 years.

**Study Information that Does Not Identify You**

You will not be named in any reports, publications, or presentations that may come from this study. Quotes obtained during the interview may be used in the dissemination of this research. Quotes will be de-identified (they have your name removed from them and only your study number will remain). and will not be linked to you. Audio recordings will be destroyed upon transcription. Prior to the analysis all transcripts will be reviewed and de-identified to ensure confidentiality.

**Voluntary participation:**

Your participation in this study is voluntary. You may decide not to be in this study, or to be in the study now, and then change your mind later. You may leave the study at any time. We will give you new information that is learned during the study that might affect your decision to stay in the study.

 You may refuse to answer any question you do not want to answer, or not answer an interview question by saying “pass”.

**Withdrawal from the study:**

If you decide to leave the study, you have the right to request withdrawal of information collected about you. If you decide to leave the study, you have the right to request withdrawal of information collected about you, including the interview information. Information can be withdrawn at anytime, even once analysis has started. Let the research team know. You may also withdraw but allow the study team to continue use of the data that has already been collected and not to collect new information. Let the research team know.

**Cost and reimbursement:**

You will not have to pay for any procedures involved in this study. As a token of appreciation, a $10 gift card to a coffee shop will be given to you.

**Rights as a participant:**

By signing this form, you do not give up any of your legal rights against the investigators, sponsor or involved institutions for compensation, nor does this form relieve the investigators, sponsor or involved institutions of their legal and professional responsibilities.

**Conflict of interest:**

Researchers have an interest in completing this study. Their interests should not influence your decision to participate in this study.

**Questions about the study:**

If you have any questions, concerns or would like to speak to the study team for any reason, please call: Dr. Katherine McGilton at 416-597-3422 ext 2500.

If you have any questions about your rights as a research participant or have concerns about this study, call the Chair of the University Health Network Research Ethics Board (UHN REB) or the Research Ethics office number at 416-581-7849. The REB is a group of people who oversee the ethical conduct of research studies. The UHN REB is not part of the study team. Everything that you discuss will be kept confidential.

You will be given a signed copy of this consent form.

**Consent:**

This study has been explained to me and any questions I had have been answered.

I know that I may leave the study at any time. I agree to the use of my information as described in this form. I agree to take part in this study.

Print Study Participant’s Name Signature Date

Print Name of Person Signature Date Obtaining Consent
