# Supplementary material for: The OASIS walking study—Older adults with cognitive impairment performing sit to stands and walking in transitional care programs: Protocol for a feasibility study
Source: PLoS One. 2024 Sep 16;19(9):e0308268. doi: 10.1371/journal.pone.0308268 (PMC11404812; doi:10.1371/journal.pone.0308268)
Supplement: S4 Appendix — (DOCX) [file pone.0308268.s007.docx]

**S4 Appendix – Evaluation to Sign Consent**

| *Is the patient alert and able to communicate?* | Yes | No |
| --- | --- | --- |

1. What are the potential risks in this study? *[risks]*
2. What is expected from you, the patient? *[study procedures]*
3. What if you don’t want to continue? *[withdrawal from the study]*
4. What if you experience discomfort? *[risks]*

**Signatures:**

I hereby certify that the patient [ Patient ID:___ ___ ] is alert, able to communicate and able to give acceptable answers to the items above.

Evaluator Signature Date
