# Supplementary material for: The OASIS walking study—Older adults with cognitive impairment performing sit to stands and walking in transitional care programs: Protocol for a feasibility study
Source: PLoS One. 2024 Sep 16;19(9):e0308268. doi: 10.1371/journal.pone.0308268 (PMC11404812; doi:10.1371/journal.pone.0308268)
Supplement: S5 Appendix — (DOCX) [file pone.0308268.s008.docx]

**S5 Appendix – Intervention Manual**

**Section 1: Overview of Intervention**

**Section 2: Human and Material Resources**

**Section 3: Procedure**

**Section 1: Overview of Intervention**

1. **Name of intervention:** The OASIS Walking Intervention

(Older Adults with cognitive impairment performing SIt to Stands and Walking)

1. **Goal of intervention:**
   1. **Ultimate goal:**
      - To increase the odds of participants being discharged back home after a transitional care program (TCP) stay
   2. **Immediate goals:**

- To improve muscle strength
- To improve mobility
- To improve functional status
- To improve quality of life

1. **Components and Activities:**
2. **Component 1:** Patient-Centred Communication Care Plan
   1. Goal: To improve communication and promote engagement and enjoyment with the intervention
   2. Activities:
      - 1. 45 minute interview with care partner (in-person or via telephone, as per care partner’s preference)
        2. 45 minute interview (30 minutes interview, 15 minutes assessment) with each participant (in-person)
        3. Creation of the Patient-Centred Communication Care Plan by the interventionist based on the two above interviews
3. **Component 2: Sit to Stand Activity**
   1. Goal: To increase participants’ lower leg muscle strength and functional status
   2. Activities: Interventionist (PI) performs Sit to stand activity with participant – sitting down and standing up with or without the use of one’s hands to reach the target for each session (target number to be discussed below), for up to 15 minutes per session, 5 sessions per week for 6 weeks; talking about participant’s interests in between the activity to make the activity more enjoyable. Research assistant (RA) stands nearby with a wheelchair.
4. **Component 2: Walking Program**
   1. Goal: To improve mobility, functional status, lower leg muscle strength, and quality of life
   2. Activities: PI walks in the TCP hallways beside the participant for up to 30 minutes per session, 5 sessions per week for 6 weeks; talking about participant’s interests during the walk to make walk more enjoyable. The RA follows closely behind the participant with a wheelchair in case the participant should get tired suddenly and need to sit down, to reduce the risk of falls.

**The figure below gives an overview OASIS Walking Study**

**Figure 1.** *Schematic of the OASIS Walking Study*


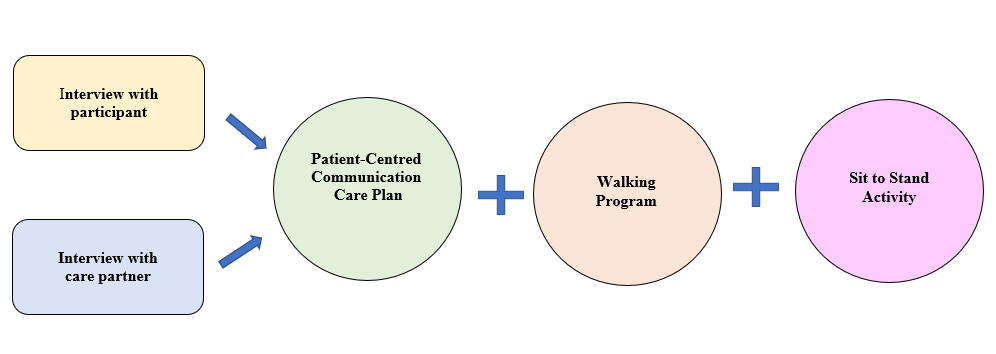


**Section 2: Human and Material Resources**

**Resources Needed:**

**Sessions 1-30 (Weeks 1-6):**

1. **Environment: Hallways in TCU**
2. **Objects:**

**For use by PI:**

- Calibrated wheel to measure distance walked
- Stopwatch to measure duration walked
- clipboard – to take notes on after the intervention session
- pen – to take notes with after the intervention
- intervention fidelity paper checklist – to complete right after the intervention session
- adherence to intervention checklist – to complete right after the intervention setting

**For use by RA:**

- wheelchair

**For use by participant:**

- Patient’s gait aid if applicable (walker, cane)
- Patient’s running shoes or other supportive footwear (help patient put these on if they do not already have them on)
- Patient’s own socks if needed (help patient put these on if they do not already have them on)
- Patient’s own hearing aid; or pocket talker from study (help patient put these on if they do not already have them on)
- Patient’s own glasses (help patient put these on if they do not already have them on)

**Section 3: Procedure**

**Each Session has an Introduction, Main Part, and Conclusion**

The OASIS Walking intervention will take place for 5 sessions per week, for 6 weeks.

Each session will last up to 45 minutes.

Up to 30 minutes is spent for the walking intervention.

Up to 15 minutes is spent for the sit to stand activity.

Details for each intervention are outlined below. Each session includes an introduction, main part, and conclusion.

| **Aspect of Intervention** | **Details** |
| --- | --- |
|  |  |
| **Introduction** | Say something such as:  “My name is _____. I am a nurse who is part of the research study on promoting walking and activity abilities.  How are you?”  Spend some time in light conversation.  The interventionist and RA spend a few minutes talking with the participant and develop rapport |
| **Main Part** | **1) Explain the Intervention:**  e.g., “Today, we will be taking a walk in the hallways for up to 30 minutes and sitting on the chair and standing up every so often (with a target of x times). Is it ok if we start now?”  **2)A) If the participant says no,**  a) Interventionist can spend a few minutes visiting if the participant allows and try to create a comfortable environment, and then ask participant if it is ok to start the intervention.  **Or**  b) Troubleshoot to make the participant more comfortable (e.g., speak to the TCU staff to assist the participant) and then approach the participant shortly after troubleshooting.  **Or**  c) Approach the participant later in the day **(for a maximum of 2 attempts in total)**  **2)B) If the participant says yes:**   - Ask the participant if they would like to walk first or do sit to stand activity first. - If walk first, pull the calibrated wheel with you to measure distance walked and start timer. - If they would like to do sit to stand first, follow the procedure in Table A and mark how many sit to stands they do. - If the participant wants to end the session early, try to encourage them to continue using the strategies in the care plan. If these are not successful, help participant back to their room.     **Table A: Procedure for Sit to Stand Activity**  (Adapted from Barreca and colleagues (44)   \| - Initially, provide instructions to the participant to promote safety. - Over time, encourage participant’s independence with sit to stand (e.g., may not need to give instructions on putting on brakes and pushing back footrests if participant remembers this instruction). - Can minimize verbal cues and hand gestures as participant improves in sit to stand performance. \| \| \| --- \| --- \| \| **Action** \| **Instruction** \| \| **If standing up from a wheelchair:** \| \| \| 1. Brakes on \| “Please put on your brakes” \| \| 2. Footrests out of the way \| “Please push your footrests back” \| \| 3. Client moves bottom forward \| “Please scoot/bring your bottom forward in the chair” \| \| 4. Feet shoulder width apart \| “Please put your feet apart” \| \| 5. Toes under the knees \| “Toes under knees” \| \| 6. Left hand on arm rest of wheelchair, right hand on walker or gait aid (or vice versa if the participant is left-handed) \| “Please put one hand on the armchair and one hand on your walker” \| \| 7. Nose over knees \| “Put your nose over your knees” \| \| 8. Stand up with pushing down left hand to help stand up and using leg muscles. Keep right hand on the walker/cane. \| “Push off the chair with your left hand and use your legs muscles to stand up. Keep your right hand on the walker/cane” \| \| 9. Provide praise/encouragement as needed. \| “Good job.” \| \| **If standing up from a standard armchair:** \| \| \| 1. Client moves bottom forward \| “Please scoot/bring your bottom forward in the chair” \| \| 2. Feet shoulder width apart \| “Please put your feet apart” \| \| 3. Toes under the knees \| ““Toes under knees” \| \| 4. Left hand on arm rest of chair, right hand on walker or gait aid (or vice versa if the participant is left-handed) \| “Please put one hand on the armchair and one hand on your walker” \| \| 5. Nose over knees \| “Put your nose over your knees” \| \| 6. Stand up with using left hand to help stand up and using leg muscles to help, keep right hand on the walker/cane. \| “Push off the chair with your left hand and use your legs muscles to stand up. Keep your right hand on the walker/cane” \| \| 7. Provide praise/encouragement as needed. \| “Good job.” \| |
| **Conclusion** | - **Thank the participant for their participation.** - **Say something like: “See you tomorrow for our next session. Have a good rest of the day”** - Record the total time for the session. - Record qualitative data – verbal comments and any non-verbal language (e.g., facial expressions, such as smiling) from the participant during and after the session. |
