# Supplementary material for: The OASIS walking study—Older adults with cognitive impairment performing sit to stands and walking in transitional care programs: Protocol for a feasibility study
Source: PLoS One. 2024 Sep 16;19(9):e0308268. doi: 10.1371/journal.pone.0308268 (PMC11404812; doi:10.1371/journal.pone.0308268)
Supplement: S6 Appendix — (PDF) [file pone.0308268.s009.pdf]

☐☐☐☐
Patient #

Date (YY-MM-DD): ☐☐-☐☐-☐☐

Initials of Assessor: ____

S6 Appendix. Time 1 Assessment (Demographic Questionnaire + Assessment Measures)

Demographic Questionnaire

1. What is your age?
2. What was your sex at birth?

   ☐ Female ☐ Male ☐ Prefer not to answer

3. What is your gender:

   ☐ Woman ☐ Man ☐ Other:

   ☐ Prefer not to answer

4. What is your ethnicity? Please select all that apply:

   ☐ White

   ☐ South Asian (e.g., East Indian, Pakistani, Sri Lankan)

   ☐ Chinese

   ☐ Black

   ☐ Filipino

   ☐ Arab

   ☐ Latin American

   ☐ Southeast Asian (e.g., Vietnamese, Cambodian, Loatian, Thai)

   ☐ West Asian (e.g., Iranian, Afghan)

   ☐ Korean

   ☐ Japanese

   ☐ Other group - Specify

   ☐ Prefer not to answer

5. What is your highest level of education (select one):

   ☐ Less than high school

   ☐ High School

Patient #

☐ Some postsecondary education

☐ Postsecondary certificate, diploma, or degree

☐ Prefer not to answer

6. What is your household income?

   ☐ Under $10,000

   ☐ $10,000 to $19,999

   ☐ $20,000 to $29,999

   ☐ $30,000 to $39,999

   ☐ $40,000 to $49,999

   ☐ $50,000 to $59,999

   ☐ $60,000 to $69,999

   ☐ $70,000 to $79,999

   ☐ $80,000 to $89,999

   ☐ $90,000 to $99,999

   ☐ $100,000

   ☐ Over $100,000 to $149,999

   ☐ Prefer not to answer

7. Where did you live before you came to the hospital:

   ☐ House/apartment/condominium

   ☐ Retirement home

   ☐ Other, please specify:

8. Do you live at home: ☐ alone

   ☐ with a care partner;

   relationship of care partner to participant: _______________

Patient #

Other Health Information

1. Do you have other diagnoses identified by your doctor, select all that apply:

	Emphysema, chronic bronchitis, chronic obstructive pulmonary disease (COPD), or chronic changes in lungs due to smoking
	High blood pressure or hypertension
	Diabetes, borderline diabetes, or high blood sugar
	Heart disease (including congestive heart failure or CHF)
	Angina (or chest pain due to heart disease), heart attack, or myocardial infarction
	Cancer
	Memory problem
	Dementia or Alzheimer's disease
	Osteoarthritis in the knee, hip or hands
	Rheumatoid or other type of arthritis
	Peripheral vascular disease or poor circulation in your limbs
	Stroke or CVA (cerebrovascular accident)
	Mini-stroke or TIA (Transient Ischemic Attack)
	Parkinsonism or Parkinson's disease
	Intestinal or stomach ulcers
	Bowel disorder such as Crohn's Disease, ulcerative colitis, or irritable bowel syndrome
	Glaucoma
	Macular degeneration
	Osteoporosis, sometimes called low bone mineral density, or thin, brittle, or weak bones
	Back problems, excluding fibromyalgia and arthritis
	Underactive thyroid gland (sometimes called hypothyroidism or myxedema) or overactive thyroid gland (sometimes called hyperthyroidism or Graves' disease)
	Kidney disease or kidney failure
	Other conditions: ___ ______
	Don't know
	Prefer not to answer

Admission Information (Check patient's chart)

1. Hospital admitting diagnosis (reason for coming to hospital): ________________
2. Admission to acute care date:
3. Admission to TCP unit:
    a. Length of stay in acute care prior to TCP Admission:

-continue to next page-

Patient #

Assessments

Procedure for Time to Perform One Sit to Stand

1. Place a chair against a wall to prevent it from moving during the test. The chair should be individualized to the participant's height so that their feet touch the ground.
2. The participant is seated in the middle of the chair, with back straight, feet approximately shoulder width apart and placed on the floor at an angle slightly back from the knees, with one foot slightly in front of the other to help maintain balance when standing
3. At the signal "go", the participant should rise to a full stand and then return back to the initial seated position.
4. The participant should perform the test two times as a practice.
5. The time to perform the third sit to stand is the actual measurement.

Time to perform one sit to stand:
First attempt: ____ seconds
Second attempt:_____ seconds
Third attempt: _____ seconds
Third attempt is the recorded actual measurement

Patient #

Procedure for 30-s Chair Stand

1. Place a chair against a wall to prevent it from moving during the test. The chair should be individualized to the participant's height so that their feet touch the ground.
2. The participant is seated in the middle of the chair, with back straight, feet approximately shoulder width apart and placed on the floor at an angle slightly back from the knees, with one foot slightly in front of the other to help maintain balance when standing
3. At the signal "go", the participant should rise to a full stand and then return back to the initial seated position.
4. Encourage the participant to complete as many full stands as possible within the 30-second limit.
5. Instruct the participant to be fully seated between each stand.
6. The tester should count the completion of each correct stand.
7. Ask the participant to perform one trial chair stand prior to the 30 second trial.
8. The total number of stands executed correctly within 30s (more than halfway up at the end of 30seconds is counted as a full stand). Incorrectly executed stands are not counted.

Total number of sit to stands done in 30 seconds: ________

Patient #

Testing Procedures for Two-Minute Walk Test

Procedure

Location:

- The preferred walking course is a 30-m-long, flat, straight enclosed indoor corridor with a hard surface.
- The turnaround points should be clearly marked with a cone.
- A starting line should be visible on the floor with brightly colored tape.
- Length of the walkway and number of turns the subject must make should be recorded.

Preparation:

- If repeated testing is required, it should be performed about the same time of the day to minimize within-day variability.
- Patients should sit at rest in a chair before the test.
- Patients should wear appropriate shoes for walking.
- Patients should use their usual walking aids during the test (cane, walker, etc.).
- Patients' usual medical regimen should be continued.
- A light meal is acceptable before early morning or early afternoon tests.
- Patients should not have exercised vigorously within 2 hours of beginning the test.

During testing:

- The pacer should walk half a meter behind patients so as not to disturb their walking pace and to ensure safety.
- No encouragement should be given to the patient, and the patient is not encouraged to talk during the test.
- One trial is given as a practice run for the patient, and the performance of the second trial is taken as the actual measurement.
- Rest of at least 10 minutes is given to the patients between each trial to avoid fatigue.
- Distance traveled during the walk test can be recorded using markings on the wall or on the floor, or using a distance measuring wheel by the pacer.

Instructions to the patient:

The purpose of this test is to find out how far you can walk in 2 minutes. You will start from this point and follow the corridor/path to the cone. You should pivot briskly around the cone like this (demonstrate to the patient how to go around the cone briskly) and continue back the other way without stopping. You will walk back and forth between the 2 cones. Don't run or jog. When the 2 minutes are up, I will say 'STOP.' I want you to stop where you are. If you become too short of breath or tired during the test to continue, you can stop at any time. When you feel more comfortable, you may start walking again. I will walk behind you because I don't want to influence the pace at which you are walking. You should not talk during the test, but I do want you to tell me if you develop any chest pain or tightness or if you become dizzy or light-headed during the test. Do you have any questions? Are you ready? Please begin when I say 'GO.

Patient #

At the completion of the 2MWT:

- Distance walked is recorded.

Distance Walked in 1st trial: _______________ metres
Distance Walked in 2nd trial: _______________ metres
Distance walked in 2nd trial is the recorded actual measurement.

Patient #

Cover page for Quality of Life for Alzheimer's Disease (QOL-AD)

Quality of Life Questionnaire for Alzheimer's Disease (QOL-AD)

(Interview Version for the person with dementia)

Interviewer to administer according to standard instructions.
Circle responses.

1. Physical health.	Poor	Fair	Good	Excellent
2. Energy level.	Poor	Fair	Good	Excellent
3. Mood.	Poor	Fair	Good	Excellent
4. Living situation.	Poor	Fair	Good	Excellent
5. Memory.	Poor	Fair	Good	Excellent
6. Family relations.	Poor	Fair	Good	Excellent
7. Marriage/closest personal relationship.	Poor	Fair	Good	Excellent
8. Friendships.	Poor	Fair	Good	Excellent
9. General self-esteem.	Poor	Fair	Good	Excellent
10. Ability to do tasks around the house.	Poor	Fair	Good	Excellent
11. Ability to do things for fun.	Poor	Fair	Good	Excellent
12. Finances.	Poor	Fair	Good	Excellent
13. Life as a whole.	Poor	Fair	Good	Excellent

Comments: ____
____
____

© 1996, Rebecca Logsdon, PhD; University of Washington

Quality of Life Questionnaire for Alzheimer's Disease (QOL-AD)

(Questionnaire Version for the Family Member or Caregiver)

The following items are about your relative's quality of life.

When you think about your relative's life, there are different aspects involved, some of which are listed below. Please think about each item, and rate your relative's current quality of life in each area using one of four words: **poor, fair, good, or excellent**. Please rate these items based on your relative's life **at the present time** (e.g., within the past few weeks). If you have questions about any item, please review the additional instructions provided.

Circle your responses.

1. Physical health.	Poor	Fair	Good	Excellent
2. Energy level.	Poor	Fair	Good	Excellent
3. Mood.	Poor	Fair	Good	Excellent
4. Living situation.	Poor	Fair	Good	Excellent
5. Memory.	Poor	Fair	Good	Excellent
6. Family relations.	Poor	Fair	Good	Excellent
7. Marriage/closest personal relationship.	Poor	Fair	Good	Excellent
8. Friendships.	Poor	Fair	Good	Excellent
9. General self-esteem.	Poor	Fair	Good	Excellent
10. Ability to do tasks around the house.	Poor	Fair	Good	Excellent
11. Ability to do things for fun.	Poor	Fair	Good	Excellent
12. Finances.	Poor	Fair	Good	Excellent
13. Life as a whole.	Poor	Fair	Good	Excellent

Comments:____

____

© 1996, Rebecca Logsdon, PhD; University of Washington

Patient #

Procedure for the Barthel ADL Index

Bowels

0= incontinent (or needs to be given enemata)

1 =occasional accident (once/week)

2 =continent

Bladder

0 = incontinent. or catheterized and unable to manage

1 = occasional accident (max once per 24 hours)

2 = continent (for over 7 days)

Grooming

0 = needs help with personal care

1 = independent face/hair/teeth/shaving (implements provided)

Toilet use

0 = dependent

1 = needs some help, but can do something alone

2 = independent (on and off, dressing wiping)

Feeding

0 = unable

1 =needs help cutting. spreading butter etc

2 = independent (food provided in reach)

Transfer

0 = unable - no sitting balance

1 =major help (one or two people, physical), can sit

2 =minor help (verbal or physical)

3 = independent

Mobility

Patient #

0 = immobile

1 =wheel chair independent including corners etc.

2 =Walks with help of one person (verbal or physical)

3 =independent (but may use any aid. e.g., stick)

Dressing

0 = dependent

1= needs help, but can do about half unaided

2 =independent (including buttons, zips, laces, etc.)

Stairs

0 =unable

1=needs help (verbal. physical. carrying aid)

2 = independent up and down

Bathing

0 = dependent

1 = independent (or in shower)

Total (11-20)

Score: _______

The Barthel ADL Index Guidelines

General

The Index should be used as a record of what the patient does NOT a record of what the patient could do.
