# Supplementary material for: The OASIS walking study—Older adults with cognitive impairment performing sit to stands and walking in transitional care programs: Protocol for a feasibility study
Source: PLoS One. 2024 Sep 16;19(9):e0308268. doi: 10.1371/journal.pone.0308268 (PMC11404812; doi:10.1371/journal.pone.0308268)
Supplement: S7 Appendix — (DOCX) [file pone.0308268.s010.docx]

**Patient ID: __________________**

**Adherence Checklist**

**Number of Treatment Sessions Attended and Level of Engagement with the Treatment**

**Goal Number of Sit to Stands for Weeks 1-3:**

**Goal Number of Sit to Stands for Weeks 4-6:**

| **Session #** | **Date** | **Time of Intervention Session** | **Session Attended by Patient** | | **Reason Not Attended (e.g. refused, too tired, away)** | **Duration of Walking Session (minutes)** | **Duration of Intervention Session (minutes)** | **Distance Walked (feet)** | **Distance Walked (metres)** | **# of sit to stands done** | **Goal Number of Sit to Stands** | **# of sit to stands done per session, divided by the goal number of sit to stands** |
| --- | --- | --- | --- | --- | --- | --- | --- | --- | --- | --- | --- | --- |
|  |  |  | **Yes** | **No** |  |  |  |  |  |  |  |  |
| **Week 1, Session 1** |  |  |  |  |  |  |  |  |  |  |  |  |
| **Week 1, Session 2** |  |  |  |  |  |  |  |  |  |  |  |  |
| **Week 1, Session 3** |  |  |  |  |  |  |  |  |  |  |  |  |
| **Week 1, Session 4** |  |  |  |  |  |  |  |  |  |  |  |  |
| **Week 1, Session 5** |  |  |  |  |  |  |  |  |  |  |  |  |
| **Week 2, Session 1** |  |  |  |  |  |  |  |  |  |  |  |  |
| **Week 2, Session 2** |  |  |  |  |  |  |  |  |  |  |  |  |
| **Week 2, Session 3** |  |  |  |  |  |  |  |  |  |  |  |  |
| **Week 2, Session 4** |  |  |  |  |  |  |  |  |  |  |  |  |
| **Week 2, Session 5** |  |  |  |  |  |  |  |  |  |  |  |  |
| **Week 3, Session 1** |  |  |  |  |  |  |  |  |  |  |  |  |
| **Week 3, Session 2** |  |  |  |  |  |  |  |  |  |  |  |  |
| **Week 3, Session 3** |  |  |  |  |  |  |  |  |  |  |  |  |
| **Week 3, Session 4** |  |  |  |  |  |  |  |  |  |  |  |  |
| **Week 3, Session 5** |  |  |  |  |  |  |  |  |  |  |  |  |
| **Week 4, Session 1** |  |  |  |  |  |  |  |  |  |  |  |  |
| **Week 4, Session 2** |  |  |  |  |  |  |  |  |  |  |  |  |
| **Week 4, Session 3** |  |  |  |  |  |  |  |  |  |  |  |  |
| **Week 4, Session 4** |  |  |  |  |  |  |  |  |  |  |  |  |
| **Week 4, Session 5** |  |  |  |  |  |  |  |  |  |  |  |  |
| **Week 5, Session**  **1** |  |  |  |  |  |  |  |  |  |  |  |  |
| **Week 5, Session**  **2** |  |  |  |  |  |  |  |  |  |  |  |  |
| **Week 5, Session**  **3** |  |  |  |  |  |  |  |  |  |  |  |  |
| **Week 5, Session**  **4** |  |  |  |  |  |  |  |  |  |  |  |  |
| **Week 5, Session**  **5** |  |  |  |  |  |  |  |  |  |  |  |  |
| **Week 6, Session 1** |  |  |  |  |  |  |  |  |  |  |  |  |
| **Week 6, Session**  **2** |  |  |  |  |  |  |  |  |  |  |  |  |
| **Week 6, Session**  **3** |  |  |  |  |  |  |  |  |  |  |  |  |
| **Week 6, Session**  **4** |  |  |  |  |  |  |  |  |  |  |  |  |
| **Week 6, Session**  **5** |  |  |  |  |  |  |  |  |  |  |  |  |
| **Total Number of Sessions for Patient (30, or less, if, for example, patient was discharged early)** |  |  |  |  |  |  |  |  |  |  |  |  |
| **Total Number Attended** |  |  |  |  |  |  |  |  |  |  |  |  |
| **Total Number of Sessions Missed** |  |  |  |  |  |  |  |  |  |  |  |  |
| **Percentage of Sessions Participant Attended** |  | | | | | | | | | | | |
