# Supplementary material for: The OASIS walking study—Older adults with cognitive impairment performing sit to stands and walking in transitional care programs: Protocol for a feasibility study
Source: PLoS One. 2024 Sep 16;19(9):e0308268. doi: 10.1371/journal.pone.0308268 (PMC11404812; doi:10.1371/journal.pone.0308268)
Supplement: S9 Appendix — (DOCX) [file pone.0308268.s012.docx]

**S9 Appendix.**

**Procedure for Assessing Assent and Dissent for the OASIS Walking Intervention**

**For the Data Collection Sessions:**

1) Upon arrival at the transitional care unit (TCU), the research assistant (RA) or interventionist introduces their self to the older adult participant.

2) The RA/interventionist will spend a few minutes talking with the participant and developing rapport.

3) Before commencing data collection, the RA/interventionist will ask permission from the participant to do a few brief tests as part of the research study (the brief tests are: **three sit to stands** as part of time to perform one sit to stand, with the third sit to stand as the actual measurement; **two two-minute walk tests**, with the second walk test as the actual measurement (as stated in proposal).

4) a) If the participant says **YES** or nods in agreement, then the participant is considered to have **assented** to the research study, and the RA/interventionist can proceed with the tests for data collection. Proceed to step 5) below.

4) b) If the participant says **NO**, the RA/interventionist can then:

a) Spend a few more minutes visiting with the participant if the participant allows and will try to create a comfortable environment. The RA/interventionist can then ask the participant again if it is ok to start with the intervention. If the participant **declines a second time,** the RA/interventionist is to end the conversation.

**Or**

b) Troubleshoot to make the participant more comfortable (e.g., speak to the TCU staff to assist the participant) and then approach the participant shortly after troubleshooting.

**Or**

c) Approach the participant later in the day (for a maximum of 2 attempts in total)

- If the participant declines a second time in the same day, do not approach them again and document refusal for the day. The participant should only be asked to participate a maximum of two times throughout the day to participate in the intervention.

The participant is considered to **dissent** to the research if they refuse to agree to do the data collection on two consecutive days (i.e., refused the intervention 4 times, 2 times each of the two consecutive days).

5) Continue to evaluate assent during the data collection. During the data collection, if the participant shows signs of restlessness or agitation, try to use the communications as outlined in the patient-centred communication care plan or offer a rest period as per the participant’s wishes.

a) If the strategies are effective, continue with the data collection.

b) If the strategies are ineffective within 5 minutes, begin to walk the participant back to their room and stop data collection.

6) Return the participant back to their room, ensure that they are comfortable and has a call bell within reach.

**For Intervention Sessions:**

1) Upon arrival at the transitional care unit (TCU), the interventionist and research assistant (RA) will introduce themselves to the older adult participant.

2) The interventionist and RA spend a few minutes talking with the participant and develop rapport.

3) Before commencing the intervention, the interventionist will ask permission from the participant to start the intervention (walk, do sit to stand activity).

4) a) If the participant says **YES** or nods in agreement, then the participant is considered to have **assented** to the research study, and the interventionist can proceed with the intervention. Proceed to step 5) below.

4) b) If the participant says **NO**, then the interventionist can:

a) Spend a few more minutes visiting with the participant if the participant allows and will try to create a comfortable environment. The interventionist can then ask the participant gain if it is ok to start with the intervention. If the participant **declines a second time,** the interventionist is to end the conversation.

**Or**

b) Troubleshoot to make the participant more comfortable (e.g., speak to the TCU staff to assist the participant) and then approach the participant shortly after troubleshooting.

**Or**

c) Approach the participant later in the day (for a maximum of 2 attempts in total)

Approach the participant later on in the day (for a total maximum of two attempts)

- If the participant declines a second time in the same day, do not approach them again and document refusal for the day. The participant should only be asked to participate a maximum of two times throughout the day to participate in the intervention.

The participant is considered to **dissent** to the research if they refuse to agree to do the intervention on two consecutive days (i.e., refused the intervention 4 times, 2 times each of the two consecutive days).

5) Continue to evaluate assent throughout the session. During the session, if the participant shows signs of restlessness or agitation, try to use the communications as outlined in the patient-centred communication care plan or offer a rest period as per the participant’s wishes.

a) If the strategies are effective, continue with the data collection.

b) If the strategies are ineffective within 5 minutes, begin to walk the participant back to their room and stop data collection.

6) Return the participant back to their room, ensure that they are comfortable and has a call bell within reach.
