# Supplementary material for: The OASIS walking study—Older adults with cognitive impairment performing sit to stands and walking in transitional care programs: Protocol for a feasibility study
Source: PLoS One. 2024 Sep 16;19(9):e0308268. doi: 10.1371/journal.pone.0308268 (PMC11404812; doi:10.1371/journal.pone.0308268)
Supplement: S10 Appendix — (DOCX) [file pone.0308268.s013.docx]

**STUDY PROTOCOL**

**The OASIS Walking Study - Older Adults with cognitive impairment performing Sit to Stands and Walking in transitional care programs: A feasibility Study**

**Principal Investigator Dr. Katherine S McGilton**

**KITE RESEARCH INSTITUTE**

**TORONTO REHABILITATION INSTITUTE**

**UNIVERSITY HEALTH NETWORK**

**Protocol Version #3**

**12/04/2023**

**Table of Contents**

# General Information 3

Background 3

Literature Review 4

Gap in the Literature 5

**2 Purpose of the Study**  6

**3 Research Questions 6**

Study Hypotheses 7

**4 Study Design 7**

**5 Selection of Participants 8**

**6 Description of Intervention 8**

Intervention Dose 9

Intervention Components 9

**7 Outcome Measures 11**

Data Collection 11

Sex and Gender Considerations During Data Collection 11

Primary Outcome 12

Secondary Outcomes 13

Outcome Data Collection Method 15

Measures to be taken to Minimize Bias 15

Plan for Missing Data 15

**8 Statistics 16**

Sample Size 16

Plan for Analysis 16

**9 Access to Source Documents 17**

**10 Ethics 18**

Recruitment and Consent Process 20

**11 References 21**

**List of abbreviations**

ALC, alternate level of care

CI, cognitive impairment

HAFD, hospital-associated functional decline

TCP, transitional care program

TCU, transitional care unit

BI, Barthel Index

OASIS Walking Intervention, Older Adults with cognitive impairment performing sit to stands and Walking in transitional care programs Intervention

PI, Principal Investigator

RA, Research Assistant

SDM, Substitute Decision-Maker

Client Satisfaction Questionnaire, CSQ

**Protocol Summary**

**The OASIS Walking Study - Older Adults with cognitive impairment performing SIt to Stands and Walking in a transitional care unit: a feasibility Study**

**Sample size**: n = 26 patient participants and their SDM (Total 52).

**Study population**: older adults ≥65 years admitted to one of two facility-based TCUs

**Study design**: A feasibility study using a quasi-experimental one-group repeated measures design will be conducted.

**Study duration**: December 2023-July 2024

**Study objectives**: The proposed study has two objectives:

1) To determine the feasibility of and satisfaction with the OASIS Walking Intervention in older adults with CI in two facility-based TCUs.

2) To determine the efficacy of the OASIS Walking Intervention on older adults’ muscle strength, mobility, functional status, quality of life, and discharge destination.

**Endpoints of the study**: The study is expected have direct value to older adult patients and their care partners. If shown to be efficacious, the study can also decrease care requirements for patients within the TCP and when they return home or when they are discharged to long-term care, which ultimately would be expected to help the health care system. The ultimate goal is to promote continued functioning, mobility, and improved quality of life for older Canadians.

# GENERAL INFORMATION

## Protocol title

The OASIS Walking Study - Older Adults with cognitive impairment performing SIt to Stands and Walking in transitional care programs: A feasibility Study

## Principal Investigator:

Dr. Katherine S. McGilton, PhD Supervisor, Senior Scientist, KITE – Toronto Rehabilitation Institute, University Health Network

Co-Investigators:

Dr. Tracey Colella, Scientist, PhD Co-Supervisor, KITE – Toronto Rehabilitation Institute, University Health Network

Dr. Martine Puts, University of Toronto

Research Assistant/Interventionist

Ms. Alexia Cumal, PhD Candidate, University of Toronto and PhD Trainee, KITE – Toronto Rehabilitation Institute, University Health Network

## Study Sites

Abbeylawn Retirement Home Transitional Care Unit (Operated by Bayshore Health Care), Pickering, Ontario

Cedarbrook Lodge Retirement Home Transitional Care Unit (Operated by Bayshore Health Care), Scarborough, Ontario

BACKGROUND

## Literature review

Cognitive impairment (CI), which can include dementia, delirium, and unspecified CI (1, 2), has a global prevalence of between 5.1% and 41%, and a median of 19.0% (3). For dementia in particular, the number of people living with this condition globally is expected to nearly double from 50 million in the year 2020 to 82 million in 2030, and 152 million in 2050 (4). In Canada, the number is expected to nearly triple from about 600,000 in 2020 to 1.7 million in 2050 (5). In Ontario, the number of people with dementia is anticipated to increase from over 228,000 in 2016 to over 430,000 in 2038 (6).

Older adults (≥65 years) (7) with CI are frequently hospitalized (8), with the most common admitting diagnoses being cerebrovascular disease, heart failure, pneumonia, transient ischemic attack, renal failure, septicemia, pulmonary embolism and urinary tract infections (9). While in hospital, they often experience mobility limitations (10), low activity levels (11), and hospital-associated functional decline (HAFD) (12), that is, the inability to perform usual activities of daily living due to weakness, reduced muscle strength, and reduced exercise capacity, which occurs due to bedrest, deconditioning, and acute illness during hospitalization (13).

Many of these older adults with CI who experience HAFD are subsequently transferred to facility-based transitional care programs (TCPs), which are short-term, post-acute care facilities that provide low-intensity restorative care (7). In Ontario, facility-based TCPs have been created for patients with a prolonged hospital length of stay who are labelled as Alternate Level of Care (ALC) and are unable to be discharged home; many are waiting to be discharged to a nursing home post hospitalization (14) and many experience a decline in their ability to perform activities of daily living (ADLs) (15). However, in a systematic review led by the interventionist for this present study (16), it was found that older adults with CI in facility-based TCPs experience worse outcomes than those without CI. While those with CI did improve in functional status in eight of 13 studies, a greater percentage of participants without CI experienced functional improvement compared to those with CI; as well, gains in functional status were smaller for older adults with CI compared to those without CI (16). These results suggest the need for additional supports and interventions to improve outcomes. Moreover, a smaller percentage of older adults with CI were discharged home post TCP, compared to those without CI (16). Given the growing number of older adults with CI, there is an urgent need for interventions in the TCP setting to improve outcomes for this population.

Older adults often experience HAFD (13, 17, 18), with a recent meta-analysis reporting a 30% prevalence of HAFD among hospitalized older adults (18). HAFD is problematic as it can have serious consequences for the health of older adults with CI. HAFD can lead to not only decreased mood and quality of life (19), but also to pressure injuries, falls, increased morbidity, and mortality (20). Moreover, HAFD can lead to rehospitalization, increased health care costs, and institutionalization (20).

HAFD can be explained by the pathophysiology of functional decline as described by the Cascade to Dependency (21) and functional decline secondary to muscle disuse models (22). Together, these models explain that aging-related factors (reduced muscle strength and aerobic capacity) combine with hazards of hospitalization (such as bedrails and tethers that promote immobility, inactivity, and bedrest) to result in disuse-induced functional decline (21, 22). Disuse-induced functional decline is characterized by muscle atrophy, loss of muscle strength, and functional deterioration, all of which increase the risk for admission to a nursing home (21, 22). Muscle atrophy occurs because of an imbalance between muscle protein synthesis and breakdown (22). During physical inactivity, the body is in a pro-inflammatory state, which is thought to lead to the activation of skeletal muscle proteolytic pathways (pathways which break down skeletal muscles) and a suppression of anabolic signaling pathways (pathways which promote the creation of muscle) (22).

To counteract the deconditioning and functional decline, there is a need for anabolic strategies (which build muscle) (22). Aerobic activity, such as walking, increases the levels of proteins (heat shock proteins) that facilitate protein synthesis and the repair of damaged proteins (22). Resistance training causes anabolic signaling pathways to be activated through increasing mechanical and metabolic stress and promotes muscle growth through increasing muscle protein synthesis; thus, resistance training such as raising from a chair as fast as possible, which uses the own body as resistance, can greatly increase muscle mass and strength and improve functional ability (22).

**Literature Review**

A comprehensive search of the literature involving mobility interventions led by nurses yielded: zero studies in the TCP setting; six studies with significant results involving older adults with cognitive impairment and walking interventions that were led by nurses or that could be done by nurses in the nursing home setting (23-30); and one study with significant results involving sit to stand activity in the nursing home setting (31).

In nursing homes, walking had a typical dose of 30 minutes per session (24, 25, 28-30), ranged from two (23) to seven (27) sessions per week; and duration ranged from six weeks (28) to six months (29). Overall, the most effective intervention was administered by a nurse and involved 2-4 walking sessions per week for 4 months and incorporated a person-centered communication care plan (23). This study had the highest recruitment and adherence rates, monitored intervention fidelity, and resulted in a significant improvement to all outcomes (functional mobility, activities of daily living function, and quality of life).

One study done in nursing homes involving older adults with dementia found that doing sit to stands 6 sit to stands per day resulted in maintained mobility (measured by the amount of time to perform one sit-to-stand) (p=0.01), which can also be a measure of lower extremity muscle strength (31).

**Gap in the Literature**

To the interventionist’s (AC’s) knowledge, there have been no nurse-led intervention studies done to date which combine sit to stand activity, a walking intervention, and a patient-centered communication care plan for older adults with CI in facility-based TCPs. Given the poorer outcomes for older adults with CI in facility-based TCPs, and the promising findings of sit to stand activity, walking interventions, and person-centered interventions in other inpatient settings, it is expected that a complex intervention which combines these three components will result in improved outcomes.

In order to address this gap in the literature, a feasibility study needs to be undertaken. A feasibility study is designed to assess an intervention, including optimal content, delivery, and adherence to the intervention, as stated in the new framework on complex intervention research that was commissioned by the Medical Research Council (MRC) and the National Institute of Health Research (NIHR) (32). This framework outlines that a feasibility study should be done to test the feasibility of a complex intervention in order to make decisions about progression to the next stage of evaluation (32).

# PURPOSE OF THE STUDY

The purpose of this study is to determine the feasibility of and satisfaction of participants with a novel intervention – the OASIS Walking Intervention (that is, the Older Adults with cognitive impairment performing Sit to Stands and Walking Intervention) in facility-based TCPs.

The second aim is to determine the efficacy of the OASIS Walking intervention on muscle strength, mobility, functional status, quality of life, and discharge destination.

# RESEARCH QUESTIONS

1. What is the feasibility of implementing the OASIS Walking Intervention in community-dwelling older adults with CI in facility-based TCPs, as determined by recruitment rate, retention rate, and adherence?

2. What is the satisfaction of older adults with CI with the OASIS Walking Intervention?

3. Does the OASIS Walking Intervention result in improved muscle strength, mobility, functional status, and quality of life in older adults with CI?

4. What percentage of the participants are discharged home and how many are discharged to the nursing home post intervention?

**Study Hypotheses**

- It is hypothesized that the OASIS Walking Intervention will be feasible as evidenced by a moderate recruitment rate (>50%), high retention rate (≥80%), and high adherence rate (attendance to ≥75% of all intervention sessions) (28).
- It is hypothesized that the participant satisfaction will be high (Client Satisfaction Questionnaire (CSQ) of 3 or more on all CSQ items) (33).
- It is hypothesized that the OASIS Walking Intervention will result in improved muscle strength (reduction in the time to perform one sit to stand by ≥0.87 seconds, p=0.01 (31), mobility (minimum detectable change (MDC) ≥9.1 meters on two-minute walk test (34)), functional ability (≥1 point improvement in Barthel Index (BI) considered as clinically meaningful (35)) in older adults with CI in facility-based TCPs, and quality of life (minimally clinical important difference is an increase in 3 points (36)).

# STUDY DESIGN

## Overall design

A feasibility study will be undertaken for this three-component intervention project. A feasibility study is in keeping with the MRC and NIHR framework which states that for complex interventions, a feasibility study is done to assess and refine the intervention prior to carrying out a full-scale evaluation (63). In terms of study design, a quasi-experimental one group time series design will be used.

## Stopping rules or discontinuation criteria

We will collect data on patients once they are admitted to the unit. Once they are discharged (including back to acute care, another site or back home) we will discontinue collecting data on the patient and we will keep track of their discharge disposition. If the patient after consenting decides to discontinue being in the study, we will withdraw them from the study.

## Setting

Transitional Care Unit (TCU) in Abbeylawn Retirement home in Pickering, Ontario and TCU in Cedarbrook Lodge Retirement Home in Scarborough Ontario. The Abbeylawn TCU admits patients designated as ALC and are destined to go to long-term care and has a 35-bed capacity. From December 2023-May 2024 (or, for 6 months from the actual start date of the study), patients will be enrolled in the study on an ongoing basis. Up to 8 patients will be enrolled in the study at a given time.

# SELECTION OF PARTICIPANTS

##

## Inclusion criteria

Older adults will be eligible if they meet the following criteria: 1) aged 65 years and older;

2) have cognitive impairment (dementia, delirium, cognitive impairment, or unspecified cognitive impairment) as documented in the medical record or Quick Dementia Rating Scale (QDRS) score of ≥2) (Appendix A); 3) admitted to a transitional care unit after a hospitalization; 4) can speak English; 5) has received clearance from the physiotherapist to participate in the study; 6) has received clearance from the nurse practitioner to participate in the study; 7) were community-dwelling (lived in a home or retirement home; not a nursing home) prior to hospitalization; 8) Were able to walk independently or with the assistance of one person (with or without a gait aid) prior to hospital admission; 9) is currently able to ambulate either independently or with the assistance of one person (with or without a gait aid); 10) has a care partner (family member, friend) who is willing participate in an interview about the patient for the study.

**Exclusion criteria**

Patients will be excluded if they are: 1) Palliative (having <six months prognosis as defined by Hui and colleagues (37)) as documented in the medical chart; 2) have Parkinson’s disease as documented in the medical chart (due to impairments in muscle and motor function) (23).

## Withdrawal of subjects

The patient, their SDM, or the care partner may withdraw from the study at any time. If they decide to leave the study, they will be presented with two choices: 1) the information that was collected before their withdrawal from the study will still be used to help answer the research question; 2) all information collected before their withdrawal will be destroyed. No new information will be collected without consent.

# DESCRIPTION OF INTERVENTION

**Description of the OASIS Walking Intervention**

The Older Adults with cognitive impairment performing the Sit to Stands and Walking Intervention is a nurse-led intervention that consists of three components: 1) Patient-Centered Communication Care Plan (informed by interviews with the participant and their care partner); 2) Sit to Stand Activity; and 3) Walking program (Figure 4). This intervention is grounded using a patient centered approach. An intervention manual has been created for this study (Appendix B).

Prior to the in-person sessions involving sit to stand activity and walking, the interventionist will review the participant’s medical chart and will conduct interviews with the participant and their care partner. The care partner can be their SDM, a family member, or friend, as determined by the patient or SDM. These interviews will inform the content of the patient-centered communication care plan.

**Intervention Dose.** The dose of the intervention is: up to 45 minutes per session, five sessions per week, for six weeks. Approximately up to 30 minutes will be spent walking with the participant and up to 15 minutes will be spent performing the sit-to-stand activity. Hospitalized older adults may have some difficulty reaching a moderate intensity of dose at the onset of the study. Indeed, a gradual increase in walking distance, speed, and frequency of walking sessions was used in a similar study with good effect (35). As such, for the first two weeks, intensity and frequency of dose will be gradually increased as tolerated by the participant, until the goal dose of 30 minutes, 5 days a week is reached.

**Intervention Components**

**Component 1:** Patient-Centered Communication Care Plan. An individualized, patient-centered communication care plan will be created for each participant, which will be used to promote enjoyment and engagement during the in-person walking and sit to stand activity sessions. The care plan will be informed by interviews that the interventionist will have with the participant and their care partner. The interview with the care partner will be 45 minutes long and can be done in-person or over the phone, according to the preference of the care partner. Specific questions that the interventionist will ask the care partners (Appendix C) and participants (Appendix D) have been adapted from a question guide used in a study with residents with dementia in nursing homes (35, 50). The interventionist will also ask the care partner about the participants’ physical function prior to hospitalization (Barthel Index).

The interview with the participant will be 45 minutes in duration (30 minutes for interview; 15 minutes for communication tests) and done in person in the patient’s room, lounge, or preferred seated area at the TCU as per patient preference. The participant’s care partner may be present with the participant during the participant’s interview as per the participant’s preference and care partner’s availability. During the interviews, the interventionist will ask questions about three areas of a patient-centered assessment: 1) Participant’s biography (work history, family, interests); 2) Participant’s communication abilities and preferences; 3) Engagement with the Participant (35, 58) (Appendix R).

The information gained from the interviews will be added to the patient-centered communication template (Figure 5). The individualized care plan will be used during the walking and sit to stand activity sessions to promote enjoyment (e.g., talking about participant’s interests, family and friends), engagement (e.g., talking about things that help to motivate participant to continue walking), and communication (e.g., using glasses to help with walking).

**Component 2:** Sit to Stand Activity. The procedure for the sit to stand activity, which is adapted from the sit to stand protocol used by Barreca 2004 and colleagues (44), is outlined in Table A, which is embedded in the intervention manual (Appendix B).

Target Number of Sit to Stands. The interventionist or RA will measure the number of sit to stands that a participant can do in 30 seconds at baseline. The procedure for this test is outlined in Appendix B and is adapted from the procedure used in previous study (84). Based on that number, a target number of sit to stands to do per session will be determined, based on the algorithm used in the study by Slaughter and colleagues (48). Halfway into the intervention (after 3 weeks), the target number of sit to stands will be progressed. The target number of sit to stands and the algorithm used by Slaughter et al. (48) (Personal communication with S. Slaughter January 2023) is outlined in the following formula:

Target number of sit to stands per occasion = (Number of sit to stands done in 30 seconds at baseline +1)

Number of occasions per day = 2 occasions on day shift + 2 occasions on evening shift = 4

Target number of sit to stands per day = 4 x (Number of sit to stands done in 30 seconds at baseline +1)

In the Slaughter et al. study, the adherence to the sit to stand activity was much lower than what was targeted; the average enactment was two sit to stands per day (rather than the target of four) and three sit to stands per occasion (rather than the target of five), for an average of six sit to stands done per day per participant (48).

For this study, the interventionist will meet with participants for one session per day, 5 days per week. Based on the above algorithm and taking into consideration the actual enactment (48), the target number of sit to stands per session for the present study was calculated. The final target number (as calculated by Slaughter and colleagues) will be the same as the progressed target number for the second half of this intervention study (for weeks 4-6); the final target number (as calculated by Slaughter and colleagues) will be multiplied by ½ to form the target for weeks 1-3 of the present study. The algorithm for the present study is outlined in the following formula:

**Weeks 1-3:**

Target number of sit to stands per session = ½ x [4 x (Number of sit to stands done in 30 seconds +1)]

**Weeks 4-6:**

Target number of sit to stands per session = 4 x (Number of sit to stands done in 30 seconds +1)

Table 1 outlines the calculated target numbers of sit to stands for the present study.

**Table 1. Target Numbers of Sit to Stands**

| **Number of**  **Sit to Stands**  **done in 30 seconds**  **at Baseline (n)** | **Initial Target Number**  **of Sit to Stands**  **per Intervention Session**  **(Weeks 1-3)** | **Progressed Target Number**  **of Sit to Stands**  **per Intervention Session**  **(Week 4-6)** |
| --- | --- | --- |
| 1 | 4 | 8 |
| 2 | 6 | 12 |
| 3 | 8 | 16 |
| 4 | 10 | 20 |
| 5 | 12 | 24 |
| n | ½ x [4 x (n+1)] | 4 x (n+1) |

In keeping with patient-centered principles, the participant can perform all sit to stands at the beginning of the session, at the end of the session, or throughout the session according to their preference. The number of sit to stands done will be recorded by the interventionist after each session.

**Component 3:** Walking Program. Based on the findings from the patient-centered assessment interviews as well as the performance of the participants on the 2MWT at baseline (Time 1), an individualized walking program will be carried out with participants, in a manner similar to the process done in the study by Chu and colleagues (35). The goal will be to walk up to 30 minutes each session, five days per week. The interventionist will personalize the dose duration and speed of each walking session as tolerated by the participant (35) and as assessed using the Borg Rate of Perceived Exertion (RPE) scale (85, 86).

The Borg Rate of Perceived Exertion (RPE) scale (38, 39) (Appendix I) will be used during the study as used in a previous study involving walking interventions and older adults with dementia (Personal communication with C. Chu, January 20, 2023 (35)). The RPE will be used to determine if the interventionist should continue the walk with the older adult participant, when to take breaks, or if it is time to stop. The goal will be to reach a moderate level of intensity (somewhat hard; RPE score of 13) (87) as has been used in a previous study involving an exercise intervention in older adults with CI (88). The RPE reached by each participant and the number of breaks taken at each session will also be recorded by the interventionist.

During the walk, the interventionist will engage the older adults in conversation about topics that are meaningful and interesting to them, as determined in the individualized patient-centered communication care plan (35). In keeping with patient-centered care principles (55), participants can engage in the walking program and perform sit to stands throughout the walk, or perform the sit to stands prior to the walk, or after the walk. The interventionist will use a calibrated wheel to measure the distance walked (in meters) and a stopwatch to measure walking duration (in minutes), as were used in a study involving older adults with dementia and a walking intervention (35).

# OUTCOME MEASURES

**Data Collection**

Demographic variables, including age, sex, gender, race/ethnicity, education, socioeconomic status, highest level of education, and number of co-morbidities will be collected before the start of the intervention (Appendix E). Data on hospital admission diagnosis and length of hospital stay prior to TCP admission will also be collected (Appendix E). Demographic data and outcome data at T1 will be obtained within the first month of the patient’s admission to the TCU.

Table 2 outlines the outcome measurement tools that will be used to assess the participants, time points at which they will be measured, and the type of statistical test that will be used to analyze the results. The total expected time for tests is 37 minutes at T1, and 29 minutes at T2 and 45 minutes at T3.

**Sex and Gender Considerations During Data Collection**

Participants’ sex (defined as a set of biological attributes; sex is usually categorized as female or male (90)) and gender (defined as socially constructed roles, behaviours, expressions, and identities of women, men, and gender diverse people; gender is usually conceptualized as a binary (woman and man) yet there is considerable diversity in how individuals understand, experience, and express it (40). Sex and gender will be assessed in the demographic questionnaire (Appendix E).

**Table 2. Outcome Measurement Tools, Time Points, and Statistical Tests**

| **Research Question**  **Number** | **Outcome (Tool)** | **Time Points** | | | | | | | | **Type of Statistical Test** |
| --- | --- | --- | --- | --- | --- | --- | --- | --- | --- | --- |
|  |  | **Pretest**  **Initial Assessment**  **(T1)** | **Weeks of Intervention** | | | | | | **Posttest (Immediately after 6-week intervention) (T3)** |  |
|  |  |  | **1** | **2** | **3** | **4**  **(T2)** | **5** | **6** |  |  |
| 1 | Recruitment Rate  (documentation) | √ |  |  |  |  |  |  |  | Descriptive statistics |
|  | Retention Rate  (documentation) | √ | √ | √ | √ | √ | √ | √ | √ | Descriptive statistics |
|  | Adherence  (checklist) |  | √ | √ | √ | √ | √ | √ |  | Descriptive statistics |
|  | Intervention Fidelity  (checklist) |  | √ | √ | √ | √ | √ | √ |  | Descriptive statistics |
| 2 | Satisfaction  (Client Satisfaction Questionnaire + 3 open ended questions) |  |  |  |  |  |  |  | √ | Descriptive statistics;  Content analysis |
| 3 | Lower extremity muscle strength (Time to perform one sit to stand) | √ |  |  |  | √ |  |  | √ | Means and SDs,  repeated measures ANOVA |
|  | Mobility  (2-minute walk test) | √ |  |  |  | √ |  |  | √ | Means and SDs, repeated measures ANOVA |
|  | Functional Status  (Barthel Index) | √ |  |  |  | √ |  |  | √ | Means and SDs,  repeated measures ANOVA |
|  | Quality of Life  (scale) | √ |  |  |  | √ |  |  | √ | Means and SDs,  repeated measures ANOVA |
| 4 | Discharge Destination  (Chart review) |  |  |  |  |  |  |  | √  (At the time of discharge from the TCU or within 60 days of admission to TCU, whichever comes first) | Descriptive statistics |

T1=time 1; T2=time 2; T3=time 3; ANOVA=analysis of variance; SDs=standard deviations.

**Primary Outcome**

**Feasibility.** will be measured through recruitment rate, retention rate, and adherence (41).

**1. Recruitment rate.** Recruitment rate will be calculated as the percentage of participants who enroll in the study out of the total number of eligible participants (23). Reasons for nonenrolment and ineligibility for the study will also be recorded. A recruitment rate of >50% is considered moderate (40).

**2. Retention rate.**  Retention rate will be calculated as the percentage of participants who complete the study (i.e., receive the full dose of the intervention and provided post-test outcome data) out of the number of participants who were enrolled (i.e., signed a consent form and provided baseline data) (41). A retention rate of ≥80% is considered high (40).

**3. Adherence.** Participants’ adherence will be determined by: 1) the number of treatment sessions attended; and 2) the level of engagement with the treatment (41). For this study, the planned number of sessions is five per week for 6 weeks for a total of 30 sessions. Thus, client adherence will be measured in two ways: 1) as a percentage: the number of sessions attended divided by the total number of sessions (30 sessions); and 2) a) the duration of each walking session, duration of each intervention session, and number of sit to stands done per session; and 2) b) the number of sit to stands done per session, divided by the goal number of sit to stands (see Table 1). Participant’s adherence to treatment will be documented by the interventionist using an adherence checklist created for this study (Appendix S) immediately after each intervention session. An adherence of ≥75% is considered high (28).

**Intervention fidelity.** Intervention fidelity will also be measured through the interventionist’s self-report of 12 intervention items (41). The percentage will be calculated as the number of items done divided by the 12 items on the intervention fidelity checklist. Immediately after each session, the interventionist will document the specific activities provided during the session using a structured checklist (Appendix S) created for this study, which includes the specific activities that were done during each session. Any safety events (such as falls) that occur during the intervention sessions will also be documented.

**Secondary Outcomes**

Efficacy will be assessed using measures for muscle strength, mobility, functional status, quality of life, and discharge destination. The primary outcome for efficacy will be lower extremity **muscle strength**.

**1. Lower extremity muscle strength.** Lower extremity muscle strength will be measured using the time to perform one sit-to-stand (42, 43). Time to perform one sit-to-stand has good validity and reliability. The ability to perform one sit to stand has been used in a study involving sit-to-stand activity with older adults with dementia in nursing homes (31). Repeated observations of the sit to stand test by one observer yielded correlations of 0.89 to 0.96 (44).

Older adults who are frail, such as those who are in nursing homes, must use their arms to stand and may be unable to complete more than one sit to stand (45). For a study involving sit-to-stand activity with older adults with dementia in nursing homes, participants were asked to stand from a seated position three times, with the time to complete the sit to stand measured at the third time using a stopwatch (31, 45) (personal communication with S. Slaughter January 16, 2023). Similarly, for the present study, the interventionist or RA will ask participants to stand from a seated position three times, with the time to complete the sit to stand measured at the third time using a stopwatch (Appendix E). The interventionist and the RAs will measure the time to complete the sit to stand for participants until ≥75% interrater agreement is reached (46). Then only one RA or the interventionist will collect the rest of the outcome measures data.

**2. Mobility.** Mobility will be measured using the two-minute walk test (2MWT). The 2MWT has good validity and reliability. It has been used in studies involving older adults with dementia in nursing homes (23, 34). As well, the 2MWT has been shown to provide sufficient data to objectively quantify gait measurements, similar to the six-minute walk test (47). The 2MWT has a test-retest reliability coefficient of 0.98 (0.96–0.99) and an inter-rater reliability of 0.92 (0.86–0.96) when assessed in frail older adults with dementia (34). Distances walked for the 2MWT among older adults with dementia in nursing homes and day care centers ranged between 63.1-63.6 meters (34). The minimal detectable change (MDC), that is, the minimum change that is considered a true change in performance, for the 2MWT is 9.1 meters (34).

The testing procedure for the 2MWT to be used will be similar to the ones used in studies involving older adults with cognitive impairment in nursing homes (34, 48) (Appendix E).

**3. Functional status.** Functional status will be measured by the Barthel Index (BI) (Appendix E), which has been shown to have a good reliability (kappa > 0.75) and validity for short stay older adult patients (49) and has acceptable item reliability (1.0) and person reliability (0.88) in older adults with dementia (50). BI is a 10-item questionnaire which is scored out of 20, with higher scores meaning a better outcome. A change in BI score of 1 point is considered a meaningful change in a person’s level of independence (35). When the BI score is multiplied by 5, a score of 0-20 indicates total dependency, 21-60 indicates severe dependency, 61-90 indicates moderate dependency, and 91-99 indicates slight dependency (51). BI scores will be measured at pretest, time 2, and post-test.

**4. Quality of life.** Quality of life will be measured using the QOL-AD Quality of Life–Alzheimer’s Disease, a 13-item questionnaire that asks about physical health, energy, mood and other quality of life measures that can be answered by older adults with CI (52). The QOL-AD has good reliability (α from 0.83 to 0.90) and validity correlation with measures of depression (r = −0.41 to −0.65) (52). A study involving a walking program for older adults with CI in nursing homes resulted in significant improvements to self-reported QOL-AD (27). It takes about 10 minutes to complete the QOL-AD (53). The QOL-AD will be completed by the SDM if the patient is not able to complete it.

5. Satisfaction. Participant satisfaction will be determined using the Client Satisfaction Questionnaire (CSQ) (54), with the cover page for the questionnaire tailored to the TCP setting, which includes 8 Likert scale questions, a comments section; and three open ended questions as was used in the work by Sano and colleagues (33) (Appendix G). As well, qualitative data in the form of field notes will also be taken on interventionists’ report of challenges, issues, and ease of intervention delivery (41). Field notes taken within the intervention fidelity checklist (Appendix S) will also capture interventionists’ perspectives on elements of the intervention that are satisfactory to participants and their care partners. The CSQ will be completed by the SDM if the patient is not able to complete it.

**5. Discharge destination.** Planned and actual discharge destinations will be determined by the interventionist through a chart review or confirmation with TCU staff.

**Additional Data Collected.** At Time 3, the RA will review the participant’s chart to document services provided to the participants in addition to the OASIS Walking Intervention, to increase the internal validity of the study. A shortened version of a checklist of services/treatments related to mobility (Table 3 below) provided to patients in transitional care program developed by McGilton and colleagues based on their scoping review on transitional care programs (7) will be completed for this study.

**Table 3: Checklist of Mobility-Related Services Provided**

| **Services** | **Yes** | **No** | **n/a** | **Comment (specify dose where appropriate)** |
| --- | --- | --- | --- | --- |
| Mobility |  |  |  | Daily/weekly/bi-weekly/other: please specify |
| Rehabilitation training including transfers, stairs, strength and balance exercises and provision of mobility aids |  |  |  | Daily/weekly/bi-weekly/other: please specify |
| Functional training in ADL (toileting, washing, dressing, walking) |  |  |  | Daily/weekly/bi-weekly/other: please specify |
| Psychosocial care measures such as central dining, recreational activities, group exercises, spiritual care |  |  |  | Daily/weekly/bi-weekly/other: please specify |

**Outcome Data Collection Method**

T1, T2, and T3 data will be recorded on paper forms by the RA or interventionist and will be entered on at least a weekly basis into a secure one drive storage database hosted in an iDAPT network for the EnCOAR Team (TRI-UHN), which has restricted access and safety backup. Data on intervention fidelity and adherence will be collected on paper forms by the interventionist immediately after each session with the participants and will be transcribed onto the one drive on a weekly basis.

**Measures to be taken to Minimize Bias**

Research assistants will be hired and will be trained to collect data from older adults and the procedures will be standardized to minimize bias. Reliable and valid measures have been selected and have well established outcome measures to minimize biases. Patients are selected to participate based on strict inclusion criteria which will minimize sampling bias.

**Plan for Missing Data**

A plan to minimize the risk of missing data will be put in place. Specifically, an Ethical Protocol and Algorithm for Data Collection and Intervention Session: Procedure for Assessing Assent and Dissent for the OASIS Walking Intervention (Appendix J) will be used. This protocol is adapted from the Ethic Protocol used by Chu and colleagues (23). For their study, all follow up assessments were completed and there was no missing data (23). This protocol includes establishing rapport with participants, obtaining assent prior to initiating data collection, and re-approaching if the participant initially refuses (Appendix J). Data on paper sheets will also be visually inspected for missing data prior to transferring them onto the secure One drive. In addition to the above retention strategy, remuneration will also be provided to reduce the risk of drop out and thus reduce the risk of missing data (see remuneration, section 7.4)

# STATISTICS

**Sample Size**

An activity-based study involving older adults with dementia in the nursing home setting (31) that used time to perform one sit to stand found a moderate effect size (Cohen’s d) of 0.48. Since the present study will use repeated measures ANOVA, a Cohen’s *f* of 0.25 (a suggested value for moderate effect size) (55) was used in the sample size calculation. Based on an *f* of 0.25, a power of 0.8 and an alpha of 0.1, in keeping with those used in a mobility study involving older adults with dementia (23), a sample of 21 participants will be needed for this study. Using an alpha of 0.1 can be acceptable for exploratory or preliminary studies (56) (p. 188). An attrition rate of just under 20% over the course of a 6-week study will be taken into account, as was experienced in a 6-week intervention study involving older adult residents with dementia (28). Thus, a sample of 26 participants will be recruited for this study. G*Power 3.1.9.7 software (57) was used for the sample size calculation for an ANOVA: Repeated measures, within factors statistical test with one group, three measurements, 0.6 for the correlation among repeated measures, and 0.8 for nonsphericity correction (which is a mild departure from sphericity) (56).

The sample size is achievable based on input provided by our collaborating TCP administrators, as the TCP has a 35-bed capacity and most patients have cognitive impairment.

## Data analysis

## Plan for Analysis

**Research question 1.** What is the feasibility of implementing the OASIS Walking Intervention in community-dwelling older adults with cognitive impairment in facility-based transitional care programs, as determined by recruitment rate, retention rate, and adherence?

Data will be analyzed using SPSS version 28.0.1.0. Descriptive statistics will be used for the demographic variables as well as for measures of feasibility (recruitment rate, retention rate, and adherence).

**Research question 2.** What is the satisfaction of older adults with CI with the OASIS Walking Intervention?

Satisfaction will be also assessed through the opened ended questions in the CSQ satisfaction surveys. Data will be analyzed using descriptive statistics. The qualitative data from the open-ended questions as well as field notes from the interventionist will be analyzed using content analysis (58).

**Research question 3.** Does the OASIS Walking intervention result in improved muscle strength, mobility, functional status, and quality of life in older adults with cognitive impairment?

Means and standard deviations for each of the outcome measures will be summarized. Longitudinal plots of overlaid individual trajectories will be used to visualize observed change over time. To analyze question 2 regarding efficacy, repeated measures analysis of variance (ANOVA) (59, 60) will be used to determine if the intervention results in an improvement in participants’ time to perform one sit to stand, 2MWT, BI, and quality of life over time. Repeated measures ANOVA is a parametric test that determines if the means of three or more measures from the same person are similar or different (60).

In order to use the test, the interventionist will check if the assumptions are met: 1) the participants are not related to one another; 2) there are at least three measures of the dependent variable; 3) there is a good range of values; 4) the dependent variable is not clearly skewed; and 5) the dependent variable is of a ratio measurement scale (60). If at least one of the assumptions is not met, the Friedman’s ANOVA by rank, a non-parametric test that is analogous to the single factor repeated-measures ANOVA (60), will be used instead. If all assumptions are met and if a significant *f* test results from the repeated measures ANOVA, the modified Bonferroni test (a more conservative test that is good to use with a smaller number of comparisons), will be used to determine which means are significantly different from each other (60). Scatter plots will be used to demonstrate any changes in outcomes. If there is missing data, paired t-tests will be used to determine if there is an improvement in participants’ time to perform one sit to stand, 2MWT, BI, and quality of life between two time points.

**Research question 4.** What percentage of the participants were discharged home and how many were discharged to the nursing home post intervention? Percentages will be used to describe discharge destination of participants.

**Sex and Gender Considerations in Analysis**

Descriptive statistics will be used to report on sex and gender. Specifically, descriptive statistics will be used to report data that is disaggregated by sex and gender.

# ACCESS TO SOURCE DOCUMENTS

Data will only be accessed by the research staff for the purpose of the study. These individuals will complete privacy training and signed confidentiality agreements and/or will be required by law to keep all collected information confidential.

Representatives of the University Health Network (UHN) including the UHN Research Ethics Board may be given remote access to an electronic portal (via the internet) to look at the study records to check that the information collected for the study is correct and to make sure the study is following proper laws and guidelines.

The electronic data will be kept in a secure one drive storage database hosted in an iDAPT network for the EnCOAR Team (TRI-UHN), which has restricted access and safety backup.

**10 Ethics**

When older adults have met eligibility criteria, the interventionist will go through the Evaluation to Sign Consent (ESC) Measure (Appendix M) (61) with them to determine their ability to sign the consent form. The ESC is normally a five-question questionnaire (four-questions for studies without random assignment), with evidence of validity and inter-rater reliability (r = .81), that has been used in nursing home residents with mild to moderate cognitive impairment to determine if they are able to sign a consent form for participation in a research study (61). The questions relate to information about the study that have been discussed during the consent process; the answers are determined by the research team and are based on the information on the consent form. If the older adults are able to answer all four questions correctly, they will be able to sign the consent form; if they do not answer all four correctly but agree to the study, proxy consent will be obtained (61). The consent forms for this study can be found in Appendices H and I.

**Assent and Dissent.** Assent can be defined as agreement to participate in research when there is less than full understanding, while dissent can be defined as refusal to participate in research even when there is proxy consent (62). Prior to each intervention session, assent from the older adults with CI will be obtained. As recommended by Slaughter and colleagues (62), a project-specific protocol for assent and dissent has been adapted from previous studies (62, 63) and developed for this study and will be used to ensure that the participants assent to research participation (Appendix J). Prior to conducting the study, ethics approval from the Research Ethics Boards at University Health Network and the University of Toronto will be obtained.

**Risks and Benefits.**

**Potential risks**

Interventions which aim to improve mobility come with a risk for falls. However, in studies involving mobility programs which were shown to improve outcomes (23, 28, 64), there were either no falls (23, 28) or no significant change in patient falls compared to the control group (64). For this study, precautions to reduce the risk of falls will be taken. In addition to the interventionist, who will walk beside the participants during the intervention, there will be a research assistant (a student in the health sciences field), who will follow closely behind the participant with a wheelchair in case of a sudden need for the participant to sit down, for the first week of the intervention and longer if indicated by the physiotherapist in the physiotherapist clearance form. This technique has been used in a previous study to reduce the risk of falls (65) (personal communication with T. Colella January 20, 2023). Other potential risks include soreness of leg muscles and a feeling of exertion are possible risks of this intervention; these symptoms have been experienced by some participants in a similar intervention study (28). For the first two weeks of the intervention, the intensity and frequency of dose will be gradually increased by the interventionist as tolerated by the participant, until the goal dose of 30 minutes for walking, 5 days a week is reached. The interventionist will also monitor the participants’ level of exertion using the RPE during the intervention sessions and will encourage rest breaks if participants express feelings of exertion. Also, as a safety precaution, only participants who have received clearance from both the physiotherapist and the nurse practitioner will be included in the study.

**Potential Benefits.**

It is hypothesized that the OASIS Walking Intervention will result in improved muscle strength, mobility, functional ability, and quality of life in older adults with CI in facility-based transitional care programs.

**Remuneration**

Participants will not have to pay for any procedures involved in this study. As a token of appreciation and in recognition of their time and effort, a $5 gift card to a coffee shop will be given to participants at each of the three outcome measurement stages of the research: 1) After the admission interview and assessment (time 1); 2) After the outcome assessment at Week 4 of the study (time 2); and 3) After the outcome assessment at the end of the study (time 3). By the end of the study, participants will be given a total of $15 in gift cards.

A $10 gift card to a coffee shop will be given to care partners (family members, close friends, substitute decision makers) of participants after their interview with the interventionist, as a token of appreciation for their time.

Providing a token gift at each stage of the research follows the guidance provided by the Division of the Vice President, Research & Innovation at the University of Toronto (66). Moreover, a recent Cochrane systematic review by Gillies and colleagues found that monetary incentives can increase participant retention in intervention studies (67).

**Recruitment and Consent Process**

**Recruitment Sites**

Participants will be recruited from the transitional care unit (TCU) in Abbeylawn Retirement Home in Pickering, Ontario and from the TCU in Cedarbrook Lodge Retirement Home in Scarborough, Ontario. Based on initial discussions with staff from the site, the units frequently admit patients with CI. Thus, it is expected that there will be sufficient eligible participants to meet the targeted sample size. Recruitment will take place over six months, in keeping with feasibility studies involving older adults with dementia (68, 69).

**Recruitment and Consent Process**

Patients: Eligible patients will be informed of the study by the TCP manager, admitting clerk, or designated staff member and ask if they are willing to have the research personnel to contact them to tell them more about the study. The interventionist (AC) will confirm eligibility, obtain consent and enroll patients into the study.

In order to confirm eligibility for the study, the interventionist will check with the unit physiotherapist (PT) and nurse practitioner (NP) to see if the patient is cleared for the study. After receiving clearance from the PT (Cedarbrook PT Clearance Form and Abbeylawn PT Clearance Form) and NP (NP Clearance Form), the interventionist will confirm with the patient and chart that they have met all eligibility criteria for the study (Appendix O – Check for Eligibility by Interventionist).

To assess the patients’ ability to provide consent, the interventionist will use a validated evaluation to sign consent (ESC) measure (Appendix M) (61). After going over and explaining the consent form, the interventionist will ask the patient to 1) identify potential risks, 2) summarize what is expected of them, the patient, 3) what they would do if they no longer want to participate in the study, 4) what if distress or discomfort associated with participating in the study is experienced. If the patient can correctly answer these questions and understand the consent form (answer in their own words), they will be asked to sign the consent form. Before asking the questions, the interventionist will make a subjective judgement whether the patient is alert and able to communicate, however if the patient no longer appears alert and able to communicate when the questions are asked, the interventionist will return to the first question, put a line through ‘yes’ answer, enter initials and date, and check no. The patient will not be considered capable to consent to participation in the study (61). For those individuals who pass the ESC, informed written consent will be obtained.

SDMs of patients who do not pass the ESC or are unable to understand the nature of the study when first approached by a circle of care representative (unit manager/ admitting clerk/ nurse/ physiotherapist / other staff member) will be contacted and informed of the study. The SDM will be asked about their interest in participation and to contact the research staff via phone or email. The interventionist will email the consent form to the SDM so that they can read more about the study and ask the interventionist if they have any questions (Appendix L). If the SDM is not comfortable with email, they can be encouraged to come to the TCU and the interventionist can give them a paper copy of the consent form and explain the study in person. The interventionist will obtain consent from the SDM to enroll the patient in the study and ask the SDM to identify a care partner familiar with the patient’s current health status and health status prior to hospitalization. Assent will be obtained from the participants prior to each intervention session.

If the care partner is identified as someone other than the SDM, they will be contacted by the SDM, informed of the study, and asked to contact the research team if interested in participating. The interventionist will obtain consent from the care partner. If the SDM identifies themselves as the care partner, they will be asked to sign two consent forms (Patient and care partner consent). Participation of care partner entails participation in an interview about the patient.

Informed consent will be obtained either in person (written consent) or electronically through REDCap. Consent will be obtained electronically only for individuals who are not able to be physically present onsite, which we anticipate being some of the SDMs or care partners. If consent is being obtained electronically, individuals will first be emailed a blank consent form for review and asked to schedule a call with the interventionist. During the call, the interventionist will review the consent form with the individual (Appendix N) and send them the REDCap link to sign the consent form (Appendix L).

# DATA HANDLING AND RECORD KEEPING

## Data Management

We will engage in systematic data management and adhere to high standards to protect participants’ confidentiality. Study data will be protected using several strategies. Participants will be given a unique identifier number. This code will not have anything to do with participants’ names. Physical copies of data will be stored in a locked cabinet at the PI’s office. Participants’ contact information, study assigned ID, signed consent forms will be stored securely and separately from completed data collection records. All data will be stored on the TRI – UHN server. The interview with participants and care partners at the beginning of the study will be audiorecorded and then transcribed. All transcripts will be anonymized, and audio recordings will be destroyed upon transcription. Upon completion of the study, data will be archived in a secure, locked location for ten years, then destroyed. In the event of inappropriate release of data, all attempts will be made to stop further release, and any information that could be retrieved will be retrieved. The UHN Privacy Office will be notified, and further actions will be taken according to the UHN Privacy Office and REB recommendations.

# REFERENCES

1. Resnick B, Beaupre L, McGilton KS, Galik E, Liu W, Neuman MD, et al. Rehabilitation Interventions for Older Individuals With Cognitive Impairment Post-Hip Fracture: A Systematic Review. Journal of the American Medical Directors Association. 2016;17(3):200-5.

2. Reynish EL, Hapca SM, De Souza N, Cvoro V, Donnan PT, Guthrie B. Epidemiology and outcomes of people with dementia, delirium, and unspecified cognitive impairment in the general hospital: prospective cohort study of 10,014 admissions. BMC Medicine. 2017;15(1):140.

3. Pais R, Ruano L, O PC, Barros H. Global Cognitive Impairment Prevalence and Incidence in Community Dwelling Older Adults-A Systematic Review. Geriatrics (Basel). 2020;5(4).

4. Alzheimer's Disease International. Numbers of people with dementia around the world. Retrieved from <https://www.alzint.org/u/numbers-people-with-dementia-2017.pdf>. 2017.

5. Alzheimer Society of Canada. Report 1 NAVIGATING THE PATH FORWARD FOR DEMENTIA IN CANADA. The Landmark Study/Path/2022. 2022.

6. Ontario Ministry of Health and Long-Term Care. Developing Ontario’s Dementia Strategy: A Discussion Paper. 2016.

7. McGilton KS, Vellani S, Krassikova A, Robertson S, Irwin C, Cumal A, et al. Understanding transitional care programs for older adults who experience delayed discharge: a scoping review. BMC geriatrics. 2021;21(1):210.

8. Shepherd H, Livingston G, Chan J, Sommerlad A. Hospitalisation rates and predictors in people with dementia: a systematic review and meta-analysis. BMC Medicine. 2019;17(1):130.

9. Zuliani G, Galvani M, Sioulis F, Bonetti F, Prandini S, Boari B, et al. Discharge diagnosis and comorbidity profile in hospitalized older patients with dementia. International journal of geriatric psychiatry. 2012;27(3):313-20.

10. Braun T, Grüneberg C, Thiel C, Schulz R-J. Measuring mobility in older hospital patients with cognitive impairment using the de Morton Mobility Index. BMC Geriatrics. 2018;18(1):100.

11. Fleiner T, Haussermann P, Mellone S, Zijlstra W. Sensor-based assessment of mobility-related behavior in dementia: feasibility and relevance in a hospital context. International Psychogeriatrics. 2016;28(10):1687-94.

12. Hartley P, Gibbins N, Saunders A, Alexander K, Conroy E, Dixon R, et al. The association between cognitive impairment and functional outcome in hospitalised older patients: a systematic review and meta-analysis. Age and ageing. 2017;46(4):559-67.

13. Kleinpell RM, Fletcher K, Jennings BM. Advances in Patient Safety

Reducing Functional Decline in Hospitalized Elderly. In: Hughes RG, editor. Patient Safety and Quality: An Evidence-Based Handbook for Nurses. Rockville (MD): Agency for Healthcare Research and Quality (US); 2008.

14. Ontario. Hallway Health Care: A System Under Strain 1st Interim Report from the Premier’s Council on Improving Healthcare and Ending Hallway Medicine. 2019.

15. Ontario. Hallway Health Care: A System Under Strain - 1st Interim Report from the Premier's Council on Improving Healthcare and Ending Hallway Medicine. 2019.

16. Cumal A, Colella TJF, Puts MT, Sehgal P, Robertson S, McGilton KS. The impact of facility-based transitional care programs on function and discharge destination for older adults with cognitive impairment: a systematic review. BMC Geriatr. 2022;22(1):854.

17. Zisberg A, Shadmi E, Gur-Yaish N, Tonkikh O, Sinoff G. Hospital-Associated Functional Decline: The Role of Hospitalization Processes Beyond Individual Risk Factors. Journal of the American Geriatrics Society (JAGS). 2015;63(1):55-62.

18. Loyd C, Markland AD, Zhang Y, Fowler M, Harper S, Wright NC, et al. Prevalence of Hospital-Associated Disability in Older Adults: A Meta-analysis. J Am Med Dir Assoc. 2020;21(4):455-61.e5.

19. Asakawa T, Koyano W, Ando T, Shibata H. Effects of Functional Decline on Quality of Life among the Japanese Elderly. The International Journal of Aging and Human Development. 2000;50(4):319-28.

20. Boltz M, Resnick B, Capezuti E, Shuluk J, Secic M. Functional Decline in Hospitalized Older Adults: Can Nursing Make a Difference? Geriatric nursing (New York). 2012;33(4):272-9.

21. Creditor MC. Hazards of hospitalization of the elderly. Ann Intern Med. 1993;118(3):219-23.

22. Valenzuela PL, Morales JS, Pareja-Galeano H, Izquierdo M, Emanuele E, de la Villa P, et al. Physical strategies to prevent disuse-induced functional decline in the elderly. Ageing Research Reviews. 2018;47:80-8.

23. Chu CH, Puts M, Parry M, Brooks D, McGilton KS. A Feasibility Study of a Multifaceted Walking Intervention to Maintain the Functional Mobility, Activities of Daily Living, and Quality of Life of Nursing Home Residents With Dementia. Rehabilitation nursing : the official journal of the Association of Rehabilitation Nurses. 2020;45(4):204-17.

24. Stevens J, Killeen M. A randomised controlled trial testing the impact of exercise on cognitive symptoms and disability of residents with dementia. Contemporary nurse : a journal for the Australian nursing profession. 2006;21(1):32-40.

25. MacRae PG, Asplund LA, Schnelle JF, Ouslander JG, Abrahamse A, Morris C. A walking program for nursing home residents: effects on walk endurance, physical activity, mobility, and quality of life. J Am Geriatr Soc. 1996;44(2):175-80.

26. Tappen RM, Roach KE, Applegate EB, Stowell P. Effect of a combined walking and conversation intervention on functional mobility of nursing home residents with Alzheimer disease. Alzheimer Dis Assoc Disord. 2000;14(4):196-201.

27. Rezola-Pardo C, Rodriguez-Larrad A, Gomez-Diaz J, Lozano-Real G, Mugica-Errazquin I, Patiño MJ, et al. Comparison Between Multicomponent Exercise and Walking Interventions in Long-Term Nursing Homes: A Randomized Controlled Trial. Gerontologist. 2020;60(7):1364-73.

28. Bossers WJ, Scherder EJ, Boersma F, Hortobágyi T, van der Woude LH, van Heuvelen MJ. Feasibility of a combined aerobic and strength training program and its effects on cognitive and physical function in institutionalized dementia patients. A pilot study. PLoS One. 2014;9(5):e97577.

29. Venturelli M, Scarsini R, Schena F. Six-Month Walking Program Changes Cognitive and ADL Performance in Patients With Alzheimer. American Journal of Alzheimer's Disease & Other Dementias®. 2011;26(5):381-8.

30. Bossers WJ, van der Woude LH, Boersma F, Hortobágyi T, Scherder EJ, van Heuvelen MJ. A 9-Week Aerobic and Strength Training Program Improves Cognitive and Motor Function in Patients with Dementia: A Randomized, Controlled Trial. Am J Geriatr Psychiatry. 2015;23(11):1106-16.

31. Slaughter SE, Wagg AS, Jones CA, Schopflocher D, Ickert C, Bampton E, et al. Mobility of Vulnerable Elders study: effect of the sit-to-stand activity on mobility, function, and quality of life. J Am Med Dir Assoc. 2015;16(2):138-43.

32. Skivington K, Matthews L, Simpson SA, Craig P, Baird J, Blazeby JM, et al. A new framework for developing and evaluating complex interventions: update of Medical Research Council guidance. Bmj. 2021;374:n2061.

33. Sano M, Egelko S, Zhu CW, Li C, Donohue MC, Ferris S, et al. Participant satisfaction with dementia prevention research: Results from Home-Based Assessment trial. Alzheimer's & dementia. 2018;14(11):1397-405.

34. Chan WLS, Pin TW. Reliability, validity and minimal detectable change of 2-minute walk test, 6-minute walk test and 10-meter walk test in frail older adults with dementia. Exp Gerontol. 2019;115:9-18.

35. Toots A, Littbrand H, Lindelöf N, Wiklund R, Holmberg H, Nordström P, et al. Effects of a High-Intensity Functional Exercise Program on Dependence in Activities of Daily Living and Balance in Older Adults with Dementia. J Am Geriatr Soc. 2016;64(1):55-64.

36. Meeuwsen EJ, Melis RJ, Van Der Aa GC, Golüke-Willemse GA, De Leest BJ, Van Raak FH, et al. Effectiveness of dementia follow-up care by memory clinics or general practitioners: randomised controlled trial. Bmj. 2012;344:e3086.

37. Hui D, Nooruddin Z, Didwaniya N, Dev R, De La Cruz M, Kim SH, et al. Concepts and definitions for "actively dying," "end of life," "terminally ill," "terminal care," and "transition of care": a systematic review. J Pain Symptom Manage. 2014;47(1):77-89.

38. Borg GA. Psychophysical bases of perceived exertion. Med Sci Sports Exerc. 1982;14(5):377-81.

39. Scherr J, Wolfarth B, Christle JW, Pressler A, Wagenpfeil S, Halle M. Associations between Borg's rating of perceived exertion and physiological measures of exercise intensity. Eur J Appl Physiol. 2013;113(1):147-55.

40. Canadian Institutes of Health Research. Definitions of Sex and Gender 2015 [July 14, 2023]. Available from: <https://cihr-irsc.gc.ca/e/47830.html>.

41. Sidani S, Braden, Carrie Jo. Design, Evluation, and Translation of Nursing Interventions. : Wiley-Blackwell; 2011.

42. Binder EF, Brown M, Craft S, Schechtman KB, Birge SJ. Effects of a Group Exercise Program on Risk Factors for Falls in Frail Older Adults. Journal of Aging and Physical Activity. 1994;2(1):25-37.

43. Bohannon RW. Sit-to-stand test for measuring performance of lower extremity muscles. Percept Mot Skills. 1995;80(1):163-6.

44. Nevitt MC, Cummings SR, Kidd S, Black D. Risk Factors for Recurrent Nonsyncopal Falls: A Prospective Study. JAMA. 1989;261(18):2663-8.

45. Slaughter SE, Estabrooks CA, Jones CA, Wagg AS. Mobility of Vulnerable Elders (MOVE): study protocol to evaluate the implementation and outcomes of a mobility intervention in long-term care facilities. BMC geriatrics. 2011;11:84.

46. Koo TK, Li MY. A Guideline of Selecting and Reporting Intraclass Correlation Coefficients for Reliability Research. J Chiropr Med. 2016;15(2):155-63.

47. Swanson CW, Haigh ZJ, Fling BW. Two-minute walk tests demonstrate similar age-related gait differences as a six-minute walk test. Gait Posture. 2019;69:36-9.

48. Connelly DM, Thomas BK, Cliffe SJ, Perry WM, Smith RE. Clinical utility of the 2-minute walk test for older adults living in long-term care. Physiother Can. 2009;61(2):78-87.

49. Ranhoff AH. Reliability of nursing assistants’ observations of functioning and clinical symptoms and signs. Aging clinical and experimental research. 1997;9(5):378-80.

50. Yi Y, Ding L, Wen H, Wu J, Makimoto K, Liao X. Is Barthel Index Suitable for Assessing Activities of Daily Living in Patients With Dementia? Front Psychiatry. 2020;11:282.

51. Physiopedia. Barthel Index 2022 [Available from: <https://www.physio-pedia.com/Barthel_Index>.

52. Logsdon RG, Gibbons LE, McCurry SM, Teri L. Assessing Quality of Life in Older Adults With Cognitive Impairment. Psychosomatic Medicine. 2002;64(3):510-9.

53. Ready RE, Ott BR. Quality of Life measures for dementia. Health Qual Life Outcomes. 2003;1:11.

54. Larsen DL, Attkisson CC, Hargreaves WA, Nguyen TD. Assessment of client/patient satisfaction: Development of a general scale. Evaluation and program planning. 1979;2(3):197-207.

55. Chen S, Chen H. Encyclopedia of Research Design. 2010 2023/03/18. Thousand Oaks

Thousand Oaks, California: SAGE Publications, Inc. Available from: <https://methods.sagepub.com/reference/encyc-of-research-design>.

56. Hertzog MA. Considerations in determining sample size for pilot studies. Res Nurs Health. 2008;31(2):180-91.

57. Faul F, Erdfelder E, Buchner A, Lang A-G. Statistical power analyses using G*Power 3.1: Tests for correlation and regression analyses. Behavior Research Methods. 2009;41(4):1149-60.

58. Hsieh H-F, Shannon SE. Three Approaches to Qualitative Content Analysis. Qualitative Health Research. 2005;15(9):1277-88.

59. Singh V, Rana RK, Singhal R. Analysis of repeated measurement data in the clinical trials. J Ayurveda Integr Med. 2013;4(2):77-81.

60. Plichta SB, Kelvin EA, Munro BH. Munro's statistical methods for health care research. 6th ed. / Stacey Plichta Kellar, Elizabeth A. Kelvin. ed. Philadelphia, Pa. ;: Wolters Kluwer, Lippincott Williams & Wilkins; 2013.

61. Resnick B, Gruber-Baldini AL, Pretzer-Aboff I, Galik E, Buie VC, Russ K, et al. Reliability and validity of the evaluation to sign consent measure. Gerontologist. 2007;47(1):69-77.

62. Slaughter S, Cole D, Jennings E, Reimer MA. Consent and Assent to Participate in Research from People with Dementia. Nursing ethics. 2007;14(1):27-40.

63. Chu CH-L. A Feasibility Study of a Multifaceted Walking Intervention to Maintain Functional Mobility, ADL Function, and Quality of Llife among Nursing Home Residents with Dementia [Doctoral Thesis]: University of Toronto; 2016.

64. Jones RA, Merkle S, Ruvalcaba L, Ashton P, Bailey C, Lopez M. Nurse-Led Mobility Program: Driving a Culture of Early Mobilization in Medical-Surgical Nursing. Journal of nursing care quality. 2020;35(1):20-6.

65. Daligadu J, Pollock CL, Carlaw K, Chin M, Haynes A, Thevaraajah Kopal T, et al. Validation of the Fitbit Flex in an Acute Post-Cardiac Surgery Patient Population. Physiother Can. 2018;70(4):314-20.

66. Division of the Vice-President - Research & Innovation - University of Toronto. Compensation & Reimbursement of Research Participants 2019 [March 14, 2023]. Available from: <https://research.utoronto.ca/compensation-reimbursement-research-participants>.

67. Gillies K, Kearney A, Keenan C, Treweek S, Hudson J, Brueton VC, et al. Strategies to improve retention in randomised trials. Cochrane Database of Systematic Reviews. 2021(3).

68. Farrand P, Woodford J, Llewellyn D, Anderson M, Venkatasubramanian S, Ukoumunne OC, et al. Behavioural activation written self-help to improve mood, wellbeing and quality of life in people with dementia supported by informal carers (PROMOTE): a study protocol for a single-arm feasibility study. Pilot and Feasibility Studies. 2016;2(1):42.

69. Dolatabadi E, Zhi YX, Flint AJ, Mansfield A, Iaboni A, Taati B. The feasibility of a vision-based sensor for longitudinal monitoring of mobility in older adults with dementia. Arch Gerontol Geriatr. 2019;82:200-6.
