# Supplementary material for: The OASIS walking study—Older adults with cognitive impairment performing sit to stands and walking in transitional care programs: Protocol for a feasibility study
Source: PLoS One. 2024 Sep 16;19(9):e0308268. doi: 10.1371/journal.pone.0308268 (PMC11404812; doi:10.1371/journal.pone.0308268)
Supplement: Attachment — Submitted filename: Response to Reviewers.docx [file pone.0308268.s014.docx]

**Responses to Feedback from Reviewers for Protocol Paper**

**PONE-D-24-00334**

**The OASIS Walking Study - older adults with cognitive impairment performing sit to stands and walking in transitional care programs: Protocol for a feasibility study**

**PLOS ONE**

Dear Dr. Mario Ulises Pérez-Zepeda,

Please find below two tables, one in which we respond to comments and questions regarding journal requirements, and one in which we respond to comments and questions asked by the reviewers.

Thank you.

Sincerely,

Alexia Cumal,

On behalf of the authors

**Response to Comments and Questions about Journal Requirements**

| **Journal Requirement** | **Response from Authors** |
| --- | --- |
| When submitting your revision, we need you to address these additional requirements.  1. Please ensure that your manuscript meets PLOS ONE's style requirements, including those for file naming. The PLOS ONE style templates can be found at  <https://journals.plos.org/>  plosone/s/file?id=wjVg/PLOSOne_formatting_  sample_main_body.pdf and  <https://journals.plos.org/plosone/s/file>?  id=ba62/PLOSOne_formatting_sample_  title_authors_affiliations.pdf | We have ensured that our manuscript meets PLOS ONE’s style requirements. |
| 2. In the online submission form, you indicated that individual participant data that underlie the results that come from the study in this article, after deidentification (text, tables, figures, and appendices) will be available upon request beginning 9 months and ending 36 months following results article publication to Investigators whose proposed use of the data has been approved by an independent review committee ("learned intermediary") identified for this purpose for the purpose of individual participant data meta-analysis..  All PLOS journals now require all data underlying the findings described in their manuscript to be freely available to other researchers, either 1. In a public repository, 2. Within the manuscript itself, or 3. Uploaded as supplementary information. This policy applies to all data except where public deposition would breach compliance with the protocol approved by your research ethics board. If your data cannot be made publicly available for ethical or legal reasons (e.g., public availability would compromise patient privacy), please explain your reasons on resubmission and your exemption request will be escalated for approval. | We cannot share our data publicly as this is not included in the informed consent forms for participants and we do not have research ethics board approval to share the data publicly.  In the online submission form, we had written that individual participant data that underlie the results that come from the study in this article, after deidentification (text, tables, figures, and appendices) will be available upon request beginning 9 months and ending 36 months following results article publication to Investigators whose proposed use of the data has been approved by an independent review committee ("learned intermediary") identified for this purpose for the purpose of individual participant data meta-analysis.  However, we realize that we had written this in error. It should say instead that we cannot share our data publicly as this is not included in the informed consent forms for participants and we do not have research ethics board approval to share the data publicly. |
| 3. Please include your full ethics statement in the ‘Methods’ section of your manuscript file. In your statement, please include the full name of the IRB or ethics committee who approved or waived your study, as well as whether or not you obtained informed written or verbal consent. If consent was waived for your study, please include this information in your statement as well. | We have included our full ethics statement in the methods section on page 24 lines 513-526.  Please find it below:  **“Ethics statement and dissemination**  The study has received written ethical approval from the University Health Network Research Ethics Board (Study ID 23-5543). Specifically, the approval letter stated: “The University Health Network Research Ethics Board approves the above mentioned study as it has been found to comply with relevant research ethics guidelines, as well as the Ontario Personal Health Information Protection Act (PHIPA), 2004” (p. 2). Should there be any important protocol modifications, the relevant parties (research ethics boards, investigators, trial participants, trial registry) will be notified. Informed written consent will be received from each participant in the study.” |

**Responses to Reviewers**

| Comments from Reviewers | Response from Authors |
| --- | --- |
| 1. Does the manuscript provide a valid rationale for the proposed study, with clearly identified and justified research questions?  The research question outlined is expected to address a valid academic problem or topic and contribute to the base of knowledge in the field.  Reviewer #1: Yes  Reviewer #2: Yes  Reviewer #3: Yes | Thank you. |
| 2. Is the protocol technically sound and planned in a manner that will lead to a meaningful outcome and allow testing the stated hypotheses?  The manuscript should describe the methods in sufficient detail to prevent undisclosed flexibility in the experimental procedure or analysis pipeline, including sufficient outcome-neutral conditions (e.g. necessary controls, absence of floor or ceiling effects) to test the proposed hypotheses and a statistical power analysis where applicable. As there may be aspects of the methodology and analysis which can only be refined once the work is undertaken, authors should outline potential assumptions and explicitly describe what aspects of the proposed analyses, if any, are exploratory.    Reviewer #1: Partly  Reviewer #2: Yes  Reviewer #3: Partly | Thank you. |
| 3. Is the methodology feasible and described in sufficient detail to allow the work to be replicable?   Descriptions of methods and materials in the protocol should be reported in sufficient detail for another researcher to reproduce all experiments and analyses. The protocol should describe the appropriate controls, sample size calculations, and re plication needed to ensure that the data are robust and reproducible.    Reviewer #1: Yes  Reviewer #2: Yes  Reviewer #3: Yes | Thank you. |
| 4. Have the authors described where all data underlying the findings will be made available when the study is complete?  The [PLOS Data policy](https://journals.plos.org/plosone/s/materials-and-software-sharing) requires authors to make all data underlying the findings described in their manuscript fully available without restriction, with rare exception, at the time of publication. The data should be provided as part of the manuscript or its supporting information, or deposited to a public repository. For example, in addition to summary statistics, the data points behind means, medians and variance measures should be available. If there are restrictions on publicly sharing data—e.g. participant privacy or use of data from a third party—those must be specified.    Reviewer #1: Yes  Reviewer #2: Yes  Reviewer #3: Yes | We cannot share our data publicly as this is not included in the informed consent forms for participants and we do not have research ethics board approval to share the data publicly. |
| 5. Is the manuscript presented in an intelligible fashion and written in standard English?  PLOS ONE does not copyedit accepted manuscripts, so the language in submitted articles must be clear, correct, and unambiguous. Any typographical or grammatical errors should be corrected at revision, so please note any specific errors here.    Reviewer #1: Yes  Reviewer #2: Yes  Reviewer #3: Yes | Thank you. |
| 6. Review Comments to the Author  Please use the space provided to explain your answers to the questions above and, if applicable, provide comments about issues authors must address before this protocol can be accepted for publication. You may also include additional comments for the author, including concerns about research or publication ethics.  You may also provide optional suggestions and comments to authors that they might find helpful in planning their study.  (Please upload your review as an attachment if it exceeds 20,000 characters) | Please see our responses below. |
| **Reviewer #1 Comments:** | |
| Reviewer #1: Major Revision Required This appears to be a well thought out study but the statistical method sections require extensive review.  The authors describe their design as ‘a quasi-experimental single group time series design’ (page 8, lines 162-3) in which assessments at 0, 3 and 6 weeks will be made (page 2, line 41-2). This can be described as repeated measures design with v = 1 pre- and w = 2 post-intervention assessments. Using the design criteria of 1-sided α = 0.1, power 1 − β = 0.8, δ = 0.48 then using a, v = 1 and w = 1, before-and-after design gives a sample size N = 21 as is stipulated on page 10, line 204. However, with the design chosen, that is v = 1 and w = 2 an explanation of how this becomes compatible with δ = 0.21 is required. This assumption would seem to imply that the change from baseline T1 to T2, that is T2 – T1, will be assumed the same as T3 − T1. In which case, there is an assumed plateau in values between T2 and T3 (see comment on statistical analysis below). However, it would be very useful to quote the actual methodology used for the sample size calculations not just a general reference to G*Power (page 10, lines 208-9). It seems very sensible to adjust the sample size to 26 to take account of possible attrition. | We thank you for your comments. We realize that we did not include an additional sentence in the manuscript after the sentence “G*Power 3.1.9.7 software (39) was used for the sample size calculation for an ANOVA”, which provides an explanation of how the sample size was calculated using G*Power.  It should have said:  G*Power 3.1.9.7 software (39) was used for the sample size calculation for an ANOVA : Repeated measures, within factors statistical test with one group, three measurements, 0.6 for the correlation among repeated measures, and 0.8 for nonsphericity correction (which is a mild departure from sphericity) (38). (pages 10-11, lines 239-241).  In total, the description of our sample size calculation includes the following:  **“Sample size**  An activity-based study involving older adults with dementia in the nursing home setting (29) that used time to perform one sit to stand found a moderate effect size (Cohen’s d) of 0.48. Since the present study will use repeated measures ANOVA, a Cohen’s *f* of 0.25 (a suggested value for moderate effect size) (37) was used in the sample size calculation. Based on an *f* of 0.25, a power of 0.8 and an alpha of 0.1, in keeping with those used in a mobility study involving older adults with dementia (21), a sample of 21 participants will be needed for this study. Using an alpha of 0.1 can be acceptable for exploratory or preliminary studies (38) (p. 188). An attrition rate of just under 20% over the course of a 6-week study will be taken into account, as was experienced in a 6-week intervention study involving older adult residents with dementia (26). Thus, a sample of 26 participants will be recruited for this study. G*Power 3.1.9.7 software (39) was used for the sample size calculation for an ANOVA: Repeated measures, within factors statistical test with one group, three measurements, 0.6 for the correlation among repeated measures, and 0.8 for nonsphericity correction (which is a mild departure from sphericity) (38).” (page 10-11, lines 228-241). |
| In general terms, a feasibility study is not anticipated to establish ‘statistically significant’ differences so that in this case, simple tests of before – after differences at 3 weeks and at 6 weeks for the outcome variables of interest are all that is required with the corresponding 95% confidence intervals (rather than the p-values) of principal interest. These would give the investigators some sense of the magnitude of changes one might look for in any subsequent trial. Consequently, I suggest that most of the methodology described in Page 20, lines 389-398 will not be appropriate. | We thank you for your comments. We have removed lines 389-398 and have added the following paragraph to the statistical analysis question for Research question 3 (please see manuscript page 22, lines 447-451).  “As well, simple tests of before – after (paired t-tests) will be used to determine the differences between Time 1 (baseline) and Time 2 (after 3 weeks of the intervention), and between Time 1 and Time 3 (after the 6-week intervention) for the outcome variables of interest. These tests will yield corresponding 95% confidence intervals, allowing investigators a sense of the magnitude of changes that one might look for in any subsequent trial.” (page 22, lines 447-451). |
| Note: Page 13, Table 2: Seems to suggest the T2 assessment is made at 4 not 3weeks | We thank you for this comment. To help with clarity, we have added a column right after week 3 but right before week 4 for the T2 assessment, and have put Table 2 in landscape orientation. |
| **Reviewer #2 Comments:** | |
| This study protocol paper aims to examine the feasibility and effects of nurse-led older adults with cognitive impairment performing sit-to-stands and walking in transitional care programs. While the study is meticulously planned and serves as a valuable protocol paper, I have provided comments due to several points of inquiry.  1, I'm not well-versed in the healthcare system in Ontario and Transitional Care Programs, so please correct me if I'm mistaken.  1-1, TCPs are provided for individuals who find it difficult to return home from acute care hospitals (due to factors like HADF) and need temporary placement until they can be accommodated in nursing home. Individuals with CI in TCPs are at higher risk of further HAFD, which is associated with adverse outcomes. With this understanding, I believe the sentence of HAFD in lines 77-82 is unnecessary or inserted around line 62. | 1. We thank you for your comments. TCPs are provided for individuals who have difficulty returning home from acute care hospitals and need temporary placement until: 1) they are well enough to go home; 2) are transferred to a retirement home; 3) are transferred to a nursing home. Based on the scoping review led by the senior author for this study (McGilton et al., 2021), TCPs are similar to skilled nursing facilities in the U.S.    1-1. We thank you for this suggestion. We have moved lines 77-82 to line 63. |
| 1-2, Is rehabilitation not provided in TCPs? If rehabilitation is provided, does its participation and frequency not affect OASIS Walking program adherence? | 1-2. Some rehabilitation is provided in TCPs. We have provided a description of usual care on page 11 (lines 257-261) to provide clarity: “For patients in the long-term care stream usual care consists of: one-to-one sessions 2-3 times per week of strengthening and balance exercises. For patients in the Rehab stream, usual care consists of: one-to-one sessions 5 times per week of strengthening, balance, and may include some walking and group therapy 5 times per week.”  Rehabilitation is not expected to affect the OASIS Walking program adherence, as the interventionist will perform the intervention sessions at times when the patient is not engaged in any other rehabilitation.  What is unique about this intervention is the addition of a nurse leading the walking and sit to stand intervention, so that each patient will have additional walking over and above what rehabilitation is provided to each individual patient. |
| 1-3, Are there concerns about facility placement during the intervention period? | 1-3. While there is a possibility that participants may be transferred to a nursing home facility during the intervention period, it is not expected that this would be the case as patients who are waiting for nursing home placement often wait many months in the TCUs before they are transferred to a facility.  We are not concerned with transfers to nursing home facilities as this is what may occur. However we plan to track if and when transfers to nursing homes occur, and we will also track if patients should be transferred to hospital during their TCP stay, as it will be part of determining the feasibility of the intervention. |
| 2, Regarding subject recruitment 2-1, Please specify the timing of the evaluation of Quick Dementia Rating Scale (QDRS) scores and whether it will be assessed in all older adult patients, as well as add details about the evaluators. | 2, 2-1. The evaluation of the QDRS score will be done prior to having the participants sign the informed consent form.  Yes, the QDRS will be assessed with all older adult patients.  A diagnosis of cognitive impairment is often under-reported in clinical records (35), and so the QDRS will be assessed in all older adult patients. Based on the results of the QDRS if the score is greater than or equal to 2, indicative of CI, the patient will be recruited.  The QDRS will be evaluated by the interventionist (AC), who is a Registered Nurse.  We have added the following paragraph to the Participants and recruitment section:  “A diagnosis of CI, such as dementia is often under-reported in clinical records (35), and so the second inclusion criterion of have CI (dementia, delirium, mild cognitive impairment, or unspecified CI) will be ascertained in one of two ways: 1) a diagnosis of CI in the medical record; or 2) QDRS score of ≥2. The QDRS is a rapid (3-5 minutes) 10-item questionnaire that is used in clinical research to assess the presence of CI for inclusion and inclusion into studies (36). The questionnaire is informant-based; informants can be spouses, adult children, relatives and friends, and paid caregivers (36). For this study, the informant will be the substitute decision maker (SDM) of the participant and the QDRS will be evaluated by the interventionist (AC), who is a Registered Nurse. A score of 0-1 indicates a high likelihood of normal cognition; 2-5 indicates mild cognitive impairment, 6-12 indicates mild dementia; 13-20 indicates moderate dementia; and 20-30 indicates severe dementia (37). The QDRS has good reliability (Cronbach α 0.86–0.93), demonstrates similar validity as the longer clinical dementia rating (CDR) scale to detect the presence of CI in older adults (36).” (page 9, lines 197-209). |
| 2-2, Is 'delirium' assessed based on it prior to admission to the TCU? | 2-2. Yes, delirium is assessed based on it being present prior to admission to the TCU. In the hospital setting, a CAM (Confusion Assessment Method) is often used to assess for delirium. The participant would have a diagnosis of delirium documented in their medical chart. |
| 2-3, What does 'cognitive impairment, or unspecified cognitive impairment as documented in the medical record' entail? Does it include a diagnosis of MCI  ? | 2-3. Cognitive impairment can include a diagnosis of mild cognitive impairment, dementia, and delirium. Unspecified cognitive impairment is cognitive impairment due to undiagnosed dementia or delirium (Reynish et al., 2017).  The medical record, specifically, forms which include documentation of the patients’ diagnoses and past medical histories as well as notes from their hospital admission, are reviewed for the presence of CI for the patient.  It is possible that a diagnosis of mild cognitive impairment is not in the chart. As we discussed above, a diagnosis of CI is often under-reported in clinical records (35). Since this is a feasibility study, we will be looking to see if individuals have a diagnosis of cognitive impairment in the chart. A QDRS will be done on all participants. |
| 2-4, Although adherence to the OASIS Walking programs and its effects may be influenced by the cause of hospitalization, should this not be considered in subject recruitment?" | 2-4. We will be collecting data on hospital diagnosis as well as past medical history, and had not planned to do take their diagnosis into account to do analysis given the small sample size. However, given this is a feasibility study we will look for trends regarding diagnosis and their ability to participate in the intervention once transferred to the TCP and the length of time it takes for them to do participate. |
| **Reviewer #3 Comments:** | |
| Dear authors, This protocol addresses an important evidence gap and is well-designed as a feasibility study. Key strengths include a clear rationale, novel intervention components, inclusion of appropriate feasibility and preliminary efficacy measures, and registration on ClinicalTrials.gov.  To further strengthen the manuscript, it is suggested to provide additional details on: | We thank you for your comments. Please see our responses below. |
| - the qualifications and training of those delivering the intervention | We have added the following information on page 11 (lines 245-250):  The interventionist is a Master’s prepared registered nurse with 10 years of clinical nursing experience on a General Internal Medicine Unit in an urban acute care hospital working with older adults with cognitive impairment, including walking, transferring, and communicating with these patients. The interventionist and research assistants will have received additional training from the unit physiotherapist on transfer training, walking, and performing the two-minute walk test. |
| - procedures for measuring adherence and participant satisfaction | We have included the adherence checklist as an appendix (**S7 Appendix. Adherence Checklist).**  We have also included the participant satisfaction questionnaire and 3 open ended questions in S8 Appendix - Time 3 Assessment with CSQ-8 and 3 open ended questions (please see pages 9-11 of S8 Appendix). |
| - a statistical analysis plan focused on the feasibility objectives (avoiding emphasis on hypothesis testing of efficacy given the small sample size), | We thank you for this suggestion. We have added a statistical analysis plan focused on the feasibility objectives. We have added the following to the statistical analysis plan for question 1 regarding feasibility:  “Specifically, for measures of adherence, the mean number of treatment sessions attended will be calculated and the range of treatment sessions will be provided. In terms of level of engagement with the treatment, the mean duration of each walking session, of each intervention session, and number of sit to stands done per session will be calculated. As well, the range of each of these durations will be provided. Furthermore, the mean number of sit to stands done per session, divided by the goal number of sit to stands will be calculated.” (page 21, lines 423-428) |
| - and procedures for data security. Reporting these methodological details, even in a feasibility study, will enhance the replicability, interpretation, and usefulness of the findings to inform future trials. | We have added a section in the manuscript called Procedures for Data Security and have included the following details:  “**Procedures for Data Security**  The research team will engage in systematic data management and adhere to high standards to protect participants’ confidentiality. Study data will be protected using several strategies. Participants will be given a unique identifier number. This code will not have anything to do with participants’ names. Physical copies of data will be stored in a locked cabinet at the PI’s office. Participants’ contact information, study assigned ID, signed consent forms will be stored securely and separately from completed data collection records. All data will be stored on the TRI – UHN server. The interview with participants and care partners at the beginning of the study will be audio-recorded and then transcribed. All transcripts will be anonymized, and audio recordings will be destroyed upon transcription. Upon completion of the study, data will be archived in a secure, locked location for ten years, then destroyed. In the event of inappropriate release of data, all attempts will be made to stop further release, and any information that could be retrieved will be retrieved. The UHN Privacy Office will be notified, and further actions will be taken according to the UHN Privacy Office and REB recommendations.” (page 24, lines 498-511) |
| - It is also suggested to more explicitly articulate the pre-specified criteria that will be used to evaluate feasibility and potential for success of a future definitive trial, | We have added a section on pre-specified criteria for feasibility and potential for success in a future trial:  **“Criteria to Evaluate Feasibility and Potential for Success of a Future Definitive Trial**  Feasibility of this study is being evaluated by recruitment rate, retention rate, and adherence rate. If the study has a recruitment rate of >50%, a retention rate of ≥80%, and an adherence rate of ≥75%, these will demonstrate feasibility of the study, and therefore potential for success in a future definitive trials. Moreover, if there is high satisfaction with the intervention (CSQ of 3 ore more on the CSQ-8 Questionnaire), this will provide additional evidence to support the testing of this intervention in a more definitive trial.” (page 23, lines 473-479). |
| - as well as the plan to obtain participant feedback on acceptability of the intervention and study procedures. | Acceptability of the intervention will be captured through our assessment of participant satisfaction with the program. At Time 3, we will be assessing satisfaction using the Client Satisfaction Questionnaire (CSQ) and 3 open ended questions. Patient satisfaction with the intervention would be indicative of acceptability of the intervention. In addition, the 3 open ended questions after the CSQ would provide additional rich data on acceptability of the intervention. The three questions are: 1. The thing I liked best about my experience in this program was: ; 2. What I liked about this program least was: ; and 3. If I could change one thing about the walking program, it would be: . |
| Finally, it would be valuable to clearly outline in the discussion how the results of this feasibility study will be used to make the decision whether or not to proceed with a definitive controlled trial and how they might inform modifications to the design of such a trial. | We thank you for this comment. We have added the following to the discussion section:  “The results of this feasibility study will be valuable for optimizing the design of a definitive controlled trial that could impact clinical practice in this population and setting. If the study demonstrates a recruitment rate of >50%, a retention rate of ≥80%, and an adherence rate of ≥75%, these will demonstrate feasibility, and will thus provide evidence to proceed with a definitive controlled trial. Moreover, if the study results in high satisfaction with the intervention, it will provide further evidence for potential for success in a future definitive trials. Furthermore, the open-ended questions, which includes the question, “If I could change one thing about the walking program, it would be:” which will be asked together with the satisfaction questionnaire will provide additional data which might inform modifications to the design of a trial.” (page 27, lines 575-583). |
| Including these details, along with the included trial registration number and an explicit statement about ethical approval, aligns well with the CONSORT extension guidelines for pilot and feasibility trials (available at <https://doi.org/10.1186/s40814-016-0105-8>). Following these recommendations would strengthen the completeness and transparency of the protocol.  With these revisions, the manuscript will be more robust and ready to inform the important next stage of study implementation. The results of this feasibility study will be valuable for optimizing the design of the eventual definitive controlled trial that could impact clinical practice in this population and setting. | In the Ethics statement and dissemination section (page 24, line 511), we revised the sentence to more explicitly state that we received ethics approval. We have also included the trial registration number. It now says:  **“**The study has received written ethical approval from the University Health Network Research Ethics Board (Study ID 23-5543). ” (page 24, lines 513-515).  and  “The study has been registered on Clinicaltrials.gov (NCT06150339). The study details can be found at https://clinicaltrials.gov/study/NCT06150339.” (page 25, lines 532-533). |
